# Supplementary material for: Randomised controlled trial of a novel online cognitive rehabilitation programme for children with cerebral palsy: a study protocol
Source: BMJ Open. 2019 Jun 4;9(6):e028505. doi: 10.1136/bmjopen-2018-028505 (PMC6561461; doi:10.1136/bmjopen-2018-028505)
Supplement: Supplementary file 1 [file bmjopen-2018-028505supp001.pdf]

Appendix A

SMART Study Qualitative Interview Questions.

Thank you for taking part in the SMART study. This study has been conducted as part of PhD project through the Queensland Cerebral Palsy and Rehabilitation Research Centre. The study's aim was to independently test the SMART intervention that was originally developed by educational psychologists in Ireland at Maynooth University and determine whether it is effective for children with cerebral palsy.

I would like to ask you about your involvement with SMART to learn more about how other families might engage with the program.

Would it be possible to audio record our conversation so I can prepare an accurate transcript of the discussion?

The recording will be transcribed and stored securely, using your study ID rather than name, to maintain confidentiality.

Parent Questions:

1) What did you like best about the SMART program?

Follow-up: Why did you like X the best?

2) What did you like least about the SMART program?

Follow-up: "Why did you not like X?"

3) What do you think could have been different in the SMART program?

4) Did you experience any challenges in supporting your child in doing SMART?

5) Did you notice any changes in your child after participating in SMART? Follow-up: Have you noticed any changes with regard to schoolwork?

If so, what were the changes you noticed in your child after participating in SMART?

6) Did your child enjoy participating in SMART?

7)Have you noticed any changes in your relationship with your child since participating in SMART?

8) Would you participate in this program again?

9) Would you recommend SMART to other families?

10) Are you planning on continuing to use SMART at home? If so, why? If not, why not?

10) Do you have any further comments or suggestions about using SMART?

Questions for children:

1) What did you like best about the SMART program?

Follow-up: Why did you like X the best?

2) What did you like least about the SMART program?

Follow-up: Why did you not like X?

3) What do you think could have been different in the SMART program?

4) Was there anything that made it easy to use SMART at home? Manual? Parent involvement?

4) Can you tell me about anything that made it hard for you to use SMART at home? Other activities? Time?

5) Did you notice any changes in yourself after participating in SMART? If yes, ask what changes have you noticed after participating in SMART?

6) Did you enjoy participating in SMART?

7) Would you participate in this program again?

9) Would you recommend SMART to other children?

10) Do you think you'd keep using SMART? If so, why? If not, why not?

11) Is there anything else you'd like to tell me about SMART?
