# Supplementary material for: Randomised controlled trial of a novel online cognitive rehabilitation programme for children with cerebral palsy: a study protocol
Source: BMJ Open. 2019 Jun 4;9(6):e028505. doi: 10.1136/bmjopen-2018-028505 (PMC6561461; doi:10.1136/bmjopen-2018-028505)
Supplement: Supplementary file 2 [file bmjopen-2018-028505supp002.pdf]

STANDARD INFORMED CONSENT - CHILD TO PARTICIPATE IN A RESEARCH PROJECT

Project Number

**HREC/14/QRCH/377**

Title of Project

A Randomised Controlled Trial of an Online Cognitive Rehabilitation Program for Children with Cerebral Palsy

Investigator(s)

Dr Koa Whittingham, Dr Jeanie Sheffield, Professor Roslyn Boyd, Ms Jane Wotherspoon

I (Parent/Guardian name) ______________________
voluntarily consent for my child to take part in the above titled Research Project, explained to me by
Mr/Ms/Dr/Professor ______________________
Child's Name ______________________
Address ______________________
Contact Phone Numbers ______________________

I have received a Parent/Guardian Information Statement to keep and I believe I understand the purpose, extent and possible effects of my child's involvement
I have been asked if I would like to have a family member or friend with me while the project was explained
I have had an opportunity to ask questions and I am satisfied with the answers I have received
I understand that the researcher has agreed not to reveal results of any information involving my child, subject to legal requirements
If information about this project is published or presented in any public form, I understand that the researcher will not reveal my child's identity
I understand that if I refuse to consent to my child's participation, or if I withdraw my child from the project at any time without explanation, this will not affect my child's access to the best available treatment options and care from Children's Health Queensland.
If you do not wish to participate, **it is okay to say no**
I understand I will receive a copy of this consent form

- **I consent to participate in this research project.**

YES □ **NO** □

SIGNATURE ______________________ Date ____________

I have explained the study to the parent/guardian who has signed above, and believe that they understand the purpose, extent and possible effects of their child's involvement in this study.

RESEARCHER'S SIGNATURE ______________________ Date ____________
Note: All parties signing the Consent Form must date their own signature.
