# Supplementary material for: Randomised controlled trial of a novel online cognitive rehabilitation programme for children with cerebral palsy: a study protocol
Source: BMJ Open. 2019 Jun 4;9(6):e028505. doi: 10.1136/bmjopen-2018-028505 (PMC6561461; doi:10.1136/bmjopen-2018-028505)
Supplement: Author's manuscript [file bmjopen-2018-028505.draft_revisions.pdf]

BMJ Open is committed to open peer review. As part of this commitment we make the peer review history of every article we publish publicly available.

When an article is published we post the peer reviewers' comments and the authors' responses online. We also post the versions of the paper that were used during peer review. These are the versions that the peer review comments apply to.

The versions of the paper that follow are the versions that were submitted during the peer review process. They are not the versions of record or the final published versions. They should not be cited or distributed as the published version of this manuscript.

BMJ Open is an open access journal and the full, final, typeset and author-corrected version of record of the manuscript is available on our site with no access controls, subscription charges or pay-per-view fees (http://bmjopen.bmj.com).

If you have any questions on BMJ Open's open peer review process please email info.bmjopen@bmj.com

BMJ Open

A randomised controlled trial of a novel online cognitive rehabilitation program for children with cerebral palsy: A study protocol

Journal:	*BMJ Open*
Manuscript ID	bmjopen-2018-028505
Article Type:	Protocol
Date Submitted by the Author:	11-Dec-2018
Complete List of Authors:	Wotherspoon, Jane; The University of Queensland, Queensland Cerebral Palsy and Rehabilitation Research Centre<br>Whittingham, Koa; The University of Queensland, Queensland Cerebral Palsy and Rehabilitation Research Centre<br>Boyd, Roslyn; The University of Queensland, Queensland Cerebral Palsy and Rehabilitation Research Centre<br>Sheffield, Jeanie; The University of Queensland, School of Psychology
Keywords:	Developmental neurology & neurodisability < PAEDIATRICS, Developmental Psychology, Cognitive Impairment, Cognitive Training

SCHOLARONE™
Manuscripts

A randomised controlled trial of a novel online cognitive rehabilitation program for children with cerebral palsy: A study protocol

Researchers: Ms Jane Wotherspoon[1]

Dr Koa Whittingham[1] (Chief Investigator)

Professor Roslyn N Boyd, PhD[1]

Dr Jeanie Sheffield[2]

Affiliations: [1]Queensland Cerebral Palsy and Rehabilitation Research Centre (QCPRRC), The University of Queensland

[3]School of Psychology, The University of Queensland

Correspondence to Jane Wotherspoon, Queensland Cerebral Palsy and Rehabilitation Research Centre, The University of Queensland

E-mail: j.wotherspoon@uq.edu.au ORCID ID: 0000-0002-6893-7196

For peer review only

For peer review only - http://bmjopen.bmj.com/site/about/guidelines.xhtml

Abstract

Introduction

Cerebral palsy (CP) is the most common cause of physical disability in children, with an estimated 600-700 infants born with CP in Australia each year. Cerebral palsy is typically associated with motor impairments, but nearly half of all children with CP also experience cognitive impairment, potentially impacting educational and vocational achievement. This paper reports the protocol for a randomised controlled trial of a computerised cognitive training intervention based on behavioural principles, Strengthening Mental Abilities through Relational Training (SMART). The study aims to investigate SMART's effect on fluid reasoning, executive function and academic achievement in children with CP.

Methods and Analysis

60 children with mild to moderate CP (Gross Motor Function Classification Scale – GMFCS I - III) aged between 8 and 12 will be recruited for this study. Participants will be randomly allocated to two groups: SMART cognitive training and wait list control. Families will access the program at home over a four-month period. Assessments will be administered at baseline, 20 weeks and at 40-week follow-up for retention. The primary outcome will be fluid intelligence, while academic achievement, executive function and social and emotional wellbeing will be secondary outcomes.

Ethics and Dissemination

This study has approval from the Children's Health Queensland Hospital and Health Service Research Ethics Committee (HREC/14/QRCH/377) and The University of Queensland (2017001806). If the computerised cognitive training program is found to

For peer review only - http://bmjopen.bmj.com/site/about/guidelines.xhtml

be effective, dissemination of these findings would assist children with CP by providing an accessible, cost-effective intervention that can be completed at home at the individual's own pace.

Registration Details

This trial is registered with the Australian New Zealand Clinical Trials Registry, Trial ID: ACTRN12617001550392. The study was registered prospectively on 10 November 2017 to present. Recruitment is underway and we aim to complete recruitment by June 2019, with data collection finalised by March 2020.

Strengths and limitations of this study

- This is the first study to trial the effectiveness of a computerised cognitive intervention in maximising fluid intelligence in children with cerebral palsy.
- It is the first randomised controlled trial of a computerised cognitive intervention based on relational frame theory for children with a developmental disability.
- Interventions for cognitive impairment in this population are not readily available, and if effective, this intervention would provide a cost-effective, easily accessible intervention.
- All participants will receive access to the computerised cognitive training intervention prior to the end of the study.
- No active control group is included in this study; therefore we cannot determine impact of the intervention independent of focused use of a computer program.

Introduction

For peer review only - http://bmjopen.bmj.com/site/about/guidelines.xhtml

Cerebral palsy (CP), with a prevalence of 1.4 per 1000 live births,[1] is the most common cause of physical disability in children and an estimated 600-700 infants are born with CP in Australia each year.[2] While CP is typically associated with motor impairments, research is now focusing on accompanying cognitive and executive functioning (EF) deficits and how these impact daily living.[3] It is estimated that approximately 45% of children with CP will have an intellectual impairment impacting significantly on educational achievement.[4-7] This translates to long-term difficulties associated with failure to complete formal education, obtain competitive employment and live independently.

While there is growing awareness of cognitive and EF limitations, interventions for CP are typically associated with improving physical activity, limb function, and participation in daily living activities.[8] Few interventions target cognitive function and academic abilities.[8] For children with CP who live in remote or isolated communities, access to clinic-based interventions is further limited. This study aims to trial a novel online cognitive rehabilitation program for children with CP targeted to improve intellectual functioning, EF, and educational achievement. The program - Strengthening Mental Abilities Through Relational Training (SMART)[9] - is founded upon relational frame theory, which proposes that the development of language and complex reasoning in humans rests upon our ability to derive relations between stimuli arbitrarily and without direct experience. An online program designed to train relational framing ability and potentially improve complex reasoning would be a cost-effective, accessible intervention for children with CP.

Cerebral palsy and cognitive impairment

For peer review only - http://bmjopen.bmj.com/site/about/guidelines.xhtml

Cerebral palsy refers to a group of motor disorders, originating in a non-progressive injury or disturbance to the brain.[10-12] These disturbances occur early in development, impacting the foetal or infant brain.[11] Cerebral palsy is associated with various types of brain lesions, [13] arising from many different causes, congenital and acquired, including intracranial haemorrhage, asphyxia, prematurity, low birth weight or infection.[14]

While motor impairment is a defining feature of CP, more recent definitions acknowledge the frequent comorbidities, such as vision problems, epilepsy, and cognitive and communication difficulties.[14, 15] Fewer studies have focused on cognitive impairment in cerebral palsy than on motor impairment, but the research that has been undertaken suggests a significant proportion of individuals with CP have impaired cognitive function.[7] A systematic review of rates of impairment in CP found that 49% of children with CP had an intellectual disability, defined as an IQ below 70, while 28% had a severe intellectual disability, with an IQ below 50.[13] Similarly, a population-based study of more than 1,100 individuals with CP in Australia found 45% had been recorded as having an intellectual disability, although level of impairment could not be determined for many in this sample.[7] It is recognised that although CP is a non-progressive disease, the impact of these additional impairments can exert significant influence over a child's development, impacting academic and vocational outcomes, psychological well-being and quality of life,[13] as well as general health.[7]

Fluid Intelligence

Amongst most intelligence researchers, there is broad agreement that intelligence is associated with certain abilities, such as problem solving, understanding abstract

For peer review only - http://bmjopen.bmj.com/site/about/guidelines.xhtml

ideas, or capacity for learning.[16-18] One prominent contemporary model, and one that is highly influential at present in the field of intelligence testing, is the Cattell-Horn-Carroll (CHC) theory of intelligence.[19, 20] CHC theory offers a system for classifying cognitive ability that has allowed greater consensus in the literature around what is being measured and referred to by terms such as crystallised and fluid intelligence.[20] Fluid intelligence is a measure of the capacity to solve novel problems and reason abstractly, while crystallised intelligence is a measure of comprehension and acquired knowledge.[21]

While in the past, intelligence was considered a stable attribute, more recently, it has been conceptualised as a quality open to change and development.[22] As a result, attention has turned to potential factors that could play a role in determining intelligence, with the aim of developing interventions.[23] Furthermore, by combining technological innovation with proposed models of cognitive plasticity, the possibility of accessible interventions, delivered via computers, iPads or similar devices, has emerged.

Various online cognitive training programs have been developed and many of these have targeted working memory (WM), an executive function that involves the ability to temporarily hold and manipulate information. Some researchers have hypothesised that gains in WM could transfer into gains in fluid intelligence.[24, 25] Research into this area is ongoing, and results are mixed.[24] While Jaeggi et al.[25] generated great interest in cognitive training when they reported gains in fluid intelligence after WM training,[26] numerous studies have failed to replicate such transfer effects,[27] and research continues in this field.

SMART program

For peer review only - http://bmjopen.bmj.com/site/about/guidelines.xhtml

The novel online cognitive training program to be trialled, Strengthening Mental Abilities through Relational Training (SMART), shows promise in pilot studies in helping improve children's cognitive skills (e.g., ability to learn, think, and reason).[9] SMART is a web-based cognitive training program that directly trains the relational abilities thought to be foundational to complex cognition. SMART is grounded in contextual behavioural science, specifically relational frame theory,[9] which proposes that all human language and complex cognition is underpinned by our ability to relate stimuli arbitrarily**.** That is, humans can relate stimuli in a manner that does not correspond to the physical properties of the stimuli. These relations between stimuli are called relational frames, and a number exist, such as coordination (e.g., same as), comparative (e.g., more/less), and temporal frames (e.g., before and after).[28] An example of a relational frame of more/less would be with Australian one- and two-dollar coins, where individuals can relate stimuli to learn that the two-dollar coin is worth more, even though it is physically smaller than the one-dollar coin.

Many relational frames, including spatial (e.g. under), measurement (e.g. bigger) and ordinal (e.g. first) are foundational to mathematics.[29] Repeated exposure to spatial, measurement, ordinal and other mathematics-relevant relational frames is considered a critical part of early education, helping students develop their understanding of mathematical concepts.[29]

In addition to learning relational frames, people can also learn behavioural responses through derived relations, not merely through direct experience.[9] Through derived relations, our relational framing abilities greatly enhance our capacity to learn and to interact effectively with others and our environment. As an example, a person might learn that the star Vega is closer to Earth than Canopus, but further than Sirius. They could then derive that Canopus is the furthest star from Earth of the three, even

For peer review only - http://bmjopen.bmj.com/site/about/guidelines.xhtml

though it has not been directly taught, and Canopus appears brighter than Vega. As relational framing is foundational to complex cognition, the direct training of relational framing itself has the potential to have wide-reaching effects on cognition.

A feasibility pilot study of SMART was conducted over a 9-month period with eight children aged 11-12 years old who had been experiencing educational difficulties at school.[9] Seven of the eight children showed significant increases in their intellectual functioning, as measured by the Wechsler Intelligence Scale for Children – Fourth Edition with scores improving by more than one standard deviation. The underlying cognitive skills trained in the program are required for vocabulary acquisition, mathematical reasoning, and other academic and learning skills. The program can be accessed in the client's home at their convenience via iPad, Mac or PC with Internet access and can be completed at the child's own pace. As such, it is a potentially cost-effective solution that can be delivered to children with CP who are unable to access ongoing rehabilitation services.

Specific Aims

The aim of this randomised controlled trial is to test the efficacy of a novel web-based cognitive rehabilitation program for children aged between 8 and 12 years old with mild to moderate congenital CP. Outcomes will be assessed immediately post-intervention (20 weeks) and again at 40 weeks post-intervention.

Hypotheses

The primary hypothesis to be tested is that in a randomised controlled trial for children aged 8-12 with CP:

1. Participants in the intervention group will demonstrate improved performance immediately post-intervention on a standardised test of intellectual ability when

For peer review only - http://bmjopen.bmj.com/site/about/guidelines.xhtml

compared to a waitlist control group receiving care as usual. (Wechsler Intelligence Scale for Children - Fifth Edition; WISC-V)[30, 31]

The secondary hypotheses to be tested are that the SMART intervention group will demonstrate improvements in the following outcomes:

1. Academic achievement (Wechsler Individual Achievement Test Third Edition; WIAT-III)[32]

2. Executive function (Behavior Rating Inventory of Executive Function; BRIEF)[33]

3. Social and emotional functioning (Strengths and Difficulties Questionnaire; SDQ; Behavior Assessment System for Children - Third Edition; BASC-3; Social Communication Questionnaire; SCQ;)[34-36].

4. Attention (Conners - Third edition; Conners-3)[37].

5. Quality of life (Cerebral Palsy Quality of Life - Child; CP-QOL)[38].

Methods

Study Design

The study is a randomised controlled trial design with waitlist control group, to determine the effectiveness of the SMART program for children with CP aged 8-12 years (see CONSORT flowchart in Figure 1.) After baseline assessment, participants will be randomly allocated to either the intervention or waitlist control group. The intervention group will commence the SMART program immediately and complete it over the following 16 weeks, before undergoing post-intervention assessment at 20 weeks. The waitlist control group will continue care as usual for 20 weeks before returning for a second assessment visit, at which point they will commence the SMART program.

Participants: We aim to recruit 60 children with mild to moderate CP (Gross Motor Function Classification Scale – GMFCS I - III) aged between 8 and 12 years old. All

For peer review only - http://bmjopen.bmj.com/site/about/guidelines.xhtml

children are required to have sufficient cooperation and cognitive understanding to perform the tasks and access the novel online cognitive training program, have access to the Internet at home, and be able to attend three assessment sessions in Brisbane. Children will be excluded if they have unstable epilepsy (i.e., not controlled by medication); an unstable brain injury (e.g., degenerative or metabolic condition); and/or active medical condition (e.g., chemotherapy, radiotherapy, or neurosurgical).

Recruitment: Participants will be recruited from a consent-based research database at the Queensland Cerebral Palsy and Rehabilitation Research Centre, and through the Queensland Paediatric Rehabilitation Service at the Queensland Children's Hospital.

Measurement of Outcome:

All children will undergo a comprehensive cognitive, psychoeducational, and psychosocial assessment by a psychologist at baseline (i.e., before treatment), and re-assessment at 20 weeks (after intervention for immediate group), and 40 weeks (retention for immediate group, and after intervention for control group). Children will complete the following assessments:

- *The Wechsler Intelligence Scale for Children Fifth Edition (WISC-V)* is a standardised measure of overall cognitive/intellectual functioning. The WISC-V produces a full-scale intelligence IQ score (FSIQ), along with five primary index scores – Verbal Comprehension Index (VCI), Visual-Spatial Index (VSI), Fluid Reasoning Index (FRI), Working Memory Index (WMI), and Processing Speed Index (PSI), each derived from two subtests. The WISC-V has been found to have good internal consistency (α=.96 for FSIQ and α= .86-.94 for primary index scores).[31] Interrater reliability has also been found to be acceptable (r= .98-.99 for interscorer agreement on a subset of subtests).[30]

For peer review only - http://bmjopen.bmj.com/site/about/guidelines.xhtml

- *The Wechsler Individual Achievement Test Third Edition (WIAT-III)* is a standardised measure of educational achievement. It provides measures of achievement across domains of oral language, reading, written language and mathematics. The internal consistency coefficients are good (α=.83-.95), with the exception of a single subtests (Alphabet Writing Fluency, α=.69), which is not used with the age range participating in this study.[32]
- *The Strengths and Difficulties Questionnaire (SDQ) - Child self-report*. This questionnaire measures the child's behaviour and adjustment. The SDQ is a 25-item measure with frequency of behaviour rated on a 3-point Likert scale.[34] The SDQ produces five subscales: emotional symptoms, conduct problems, inattention/hyperactivity, peer problems and prosocial behaviour (range 0-10), and a total difficulties score (range 0-40). Research suggest that for younger children, internal consistency is acceptable for the total difficulties, emotional symptoms, prosocial behaviour, and inattention/hyperactivity subscales, but not for peer and conduct problems.[39] However, Muris et al.[39] note that the scale can provide useful information about psychopathology in children from eight years old.

For peer review only

Parents will also be administered questionnaires during the three assessments points. These include:

- *The Behaviour Rating Inventory of Executive Function (BRIEF)* assesses the child's executive functioning in everyday life. The *BRIEF* is an 85-item parent-rated questionnaire assessing behavioural manifestations of executive functions in everyday life.[33] The *BRIEF* produces a Behavioural Regulation Index (BRI; Initiate, Working Memory, Plan/Organise, Organisation of Materials, and Monitor subscales) a Metacognition Index (MCI; Inhibit,

For peer review only - http://bmjopen.bmj.com/site/about/guidelines.xhtml

Emotional Control, and Shift subscales). The *BRIEF* has been shown to be a valid measure of executive functioning and has good internal consistency (α=.80-.98) and high test-retest reliability on the BRI (r=.92), MCI (r=.88), and the GEC (r=.86).

- *Conners-3 Parent Report,* which assesses childhood behaviours and behavioural disorders including Attention Deficit Hyperactive Disorder in children and adolescents aged 6-18.[37] The Conners-3 consists of 110 statements and takes approximately 20 minutes to complete. The Conners-3 measures seven key areas – inattention, learning problems, aggression, family relations, hyperactivity/impulsivity, executive functioning, and peer relations. Both internal consistency coefficients (α=.83-.94) and test-retest reliability (r=.52-.94) are good.
- The *Behavior Assessment System for Children – 3rd Edition (BASC-3)* measures children's adaptive and problem behaviours and emotional difficulties at home and in community settings [36]. It is completed by a parent or guardian and consists of 175 statements, taking approximately 20 minutes. The *BASC-3* produces five composite scales, Internalizing Problems, Externalizing Problems, School Problems, Adaptive Skills and the Behavioural Symptoms Index. Internal consistency coefficients for the Parent Rating Scale (α=.79-.97 for 6- to 7-year-olds; α=.79-.96 for 8- to 11-year-olds) and test-retest reliability (r=.80-.92 for Child scales) are good.
- *Social Communication Questionnaire – Current, (SCQ - Current)* screens for communication skills and social functioning difficulties associated with autism spectrum disorders. The *SCQ – Current* evaluates communication skills and social functioning in children, based on the previous three months.

For peer review only - http://bmjopen.bmj.com/site/about/guidelines.xhtml

While research suggests the SCQ – Current has lower internal consistency than the Lifetime version,[40] concerns around its use particularly focus on children below five years of age, which is outside the age range of participants in this study, and on using the Current form as an alternative to the Lifetime form for screening for Autism Spectrum Disorder. For the purpose of measuring change over time, however, the Current form is recommended.[35] It contains 40 items and is completed by a parent or primary caregiver in under 10 minutes. Scores on the SCQ provide information on the following domains: Social Interaction, Communication and Stereotyped, Repetitive or Restricted Behaviour.

- *Strengths and Difficulties Questionnaire (SDQ) – Parent Report* measures pro-social, internalising and externalising behaviours in children. It is a 25-item measure with frequency of behaviour rated on a 3-point Likert scale. The *SDQ* is a 25-item parent-report measure of child behaviour and adjustment, with frequency of behaviour rated on a 3-point Likert scale.[34] The SDQ produces five subscales: emotional symptoms, conduct problems, inattention/hyperactivity, peer problems and prosocial behaviour (range 0-10). It produces a total difficulties score (range 0-40) found to have adequate internal reliability (α=.73) and test-retest reliability (r=.62) as well as discriminant and concurrent validity.[34]

- *Cerebral Palsy Quality of Life for Children- Parent Report (CP-QOL-Child)* measures child quality of life. *CP QOL-Child* is a 66-item parent-report measure of child quality of life specifically developed for use in children with CP.[38] It measures quality of life across domains including physical wellbeing,

For peer review only - http://bmjopen.bmj.com/site/about/guidelines.xhtml

social wellbeing, emotional wellbeing, school, service access and social acceptance. It has good concurrent validity, internal consistency (α=.76-.89) and test-retest reliability (r=.80-.90).[38]

Assessments at baseline, 20 and 40 weeks will take approximately two to three hours per session. The waitlist control design ensures all children in the study will receive the intervention within six months of being randomised to either commence the program immediately or after 20 weeks.

At the conclusion of the study, semi-structured interviews will be conducted with children and caregivers to explore their engagement with the online cognitive rehabilitation program and gain qualitative insights into families' experience with the program. Questions will cover what families liked and disliked about the program, how easy they found it was to access at home, and to remain engaged. Interviews will be recorded, transcribed and analysed.

Intervention: With the SMART online platform, participants answer problems directly training relational framing and receive immediate feedback on their answers. Study participants are provided with an alias login name (to maintain confidentiality) and a password. Children are encouraged to complete the SMART intervention for 30 minutes per session, for a total of 1.5 hours per week. SMART is incremented and can be completed at the child's own pace. The full dosage is reached when the child completes the entire intervention, which is expected to occur within 20 weeks. Throughout the RCT, a psychologist will stay in contact with the family and monitor that child's progress, supporting both parent and child in meeting their goals for completing the program, and maintaining engagement.

A resource guide prepared for this study will be provided to all families. This guide provides technical information on accessing the program, a description of how to

For peer review only - http://bmjopen.bmj.com/site/about/guidelines.xhtml

work through each stage of the program, and information on how parents can support their child to work on the program, including a visual chart to keep track of progress.

Statistical Analysis: Study hypotheses will be analysed by means of appropriate statistical tests, with statistical significance for all tests set at $p<0.05$ with adjustment for multiple comparisons, and all analyses will be intention to treat. Mixed analysis of variance analyses will be conducted with time (baseline, 20 weeks and 40 weeks) as the within subjects variable, and group (intervention or waitlist) as the between subjects variable. Secondary analysis will profile cognitive change over time for participants based on their test scores. Power analysis suggests a total sample size of 52 would be sufficient to equate to a medium-large effect size of 0.70 (power 0.8).[41] We aim to recruit 60 children to account for attrition (30 in each group).

Ethics and Dissemination

This study has received full ethical approval from the Children's Health Queensland Hospital and Health Service Research Ethics Committee (HREC/14/QRCH/377) and The University of Queensland (2017001806). Protocol modifications and amendments will be submitted to the ethics committees for approval. This trial has been registered with the Australian New Zealand Clinical Trials Registry, Trial ID: ACTRN12617001550392. Study results will be disseminated through publication in scientific journals and participation in conferences. Families who participate in the study will receive information on the study results, as well as a feedback report on the outcomes of assessments their child has completed. If the computerised cognitive training program is found to be effective, dissemination of these findings would assist children with CP by providing an easily accessible, cost-effective intervention that can be completed at home at the individual's own pace.

Discussion

For peer review only

This protocol paper has reported the background and study design for a randomised controlled trial investigating the effectiveness of a computerised cognitive training program for children with CP. This program has not previously been studied in this population. The research study will assess children's cognitive skills, executive ability and social and emotional functioning, with fluid intelligence the primary outcome of interest. Qualitative information will be gathered on families' experience engaging with the program. Results of the study will be disseminated through peer-reviewed journals and at relevant scientific conferences.

Nearly half of all children with CP are estimated to also have an intellectual impairment, impacting academic achievement and ability to achieve educational and vocational goals in the long-term. If this computerised cognitive training program is found to be effective, a flexible, easily accessible intervention will be available for this population, where at present there at few options available for addressing difficulties with cognitive skills in CP.

Author Contributions

The study was designed and established by all the authors. JW is responsible for the ethics application and reporting. JW is responsible for recruitment and data collection. JW will take a lead role in preparing publications on the clinical outcomes of the study. KW, RB and JS will contribute to the preparation of publications and are providing supervision throughout the study. JW will take on a lead role for statistical analysis. JW drafted the final version of this manuscript, while all authors critically reviewed and approved the final version. JW will use data from this study to contribute to her PhD thesis.

Funding

For peer review only - http://bmjopen.bmj.com/site/about/guidelines.xhtml

JW is a PhD scholar funded by an Australian Government Research Training Program Stipend and Queensland Cerebral Palsy and Research Rehabilitation Centre Top-up Scholarship.

Competing Interests

The authors state they have no competing interests in this project.

For peer review only

For peer review only - http://bmjopen.bmj.com/site/about/guidelines.xhtml

References

1. Australian Cerebral Palsy Group. Report of the Australian Cerebral Palsy Register Birth years 1995-2012, November 2018 2018. Available from: https://www.cpregister.com/pubs/pdf/ACPR-Report_Web_2018.pdf

2. Access Economics. The economic impact of cerebral palsy in Australia in 2007. Canberra: Access Economics, 2008.

3. Bottcher L, Flachs EM, Uldall P. Attentional and Executive Impairments in Children with Spastic Cerebral Palsy. Developmental Medicine & Child Neurology. 2010;52(2):e42-e7.

4. Himmelmann K, Beckung E, Hagberg G, Uvebrant P. Gross and fine motor function and accompanying impairments in cerebral palsy. Developmental Medicine & Child Neurology. 2006;48(6):417-23.

5. Rankin J, Cans C, Garne E, Colver A, Dolk H, Uldall P, et al. Congenital anomalies in children with cerebral palsy: a population-based record linkage study. Developmental Medicine & Child Neurology. 2010;52(4):345-51.

6. Andersen GL, Irgens LM, Haagaas I, Skranes JS, Meberg AE, Vik T. Cerebral palsy in Norway: Prevalence, subtypes and severity. European Journal of Paediatric Neurology. 2008;12(1):4-13.

7. Reid SM, Meehan EM, Arnup SJ, Reddihough DS. Intellectual disability in cerebral palsy: a population-based retrospective study. Developmental Medicine & Child Neurology. 2018;60(7):687-94.

8. Novak I, McIntyre S, Morgan C, Campbell L, Dark L, Morton N, et al. A systematic review of interventions for children with cerebral palsy: state of the evidence. Developmental Medicine & Child Neurology. 2013;55(10):885-910.

For peer review only - http://bmjopen.bmj.com/site/about/guidelines.xhtml

9. Cassidy S, Roche B, Hayes SC. A relational frame training intervention to raise intelligence quotients: A pilot study. The Psychological Record. 2011;61(2):173-98.

10. Badawi N, Watson L, Petterson B, Blair E, Slee J, Haan E, et al. What constitutes cerebral palsy? Developmental Medicine & Child Neurology. 1998;40(8):520-7.

11. Rosenbaum P. The Definition and Classification of Cerebral Palsy: Are We Any Further Ahead in 2006? NeoReviews. 2006;7(11):e569-e74.

12. Smithers-Sheedy H, Badawi N, Blair E, Cans C, Himmelmann K, Krägeloh-Mann I, et al. What constitutes cerebral palsy in the twenty-first century? Developmental Medicine & Child Neurology. 2014;56(4):323-8.

13. Novak I, Hines M, Goldsmith S, Barclay R. Clinical prognostic messages from a systematic review on cerebral palsy. Pediatrics. 2012;130(5):e1285.

14. Straub K, Obrzut J. Effects of Cerebral Palsy on Neuropsychological Function. Journal of Developmental and Physical Disabilities. 2009;21(2):153-67.

15. Rosenbaum P, Paneth N, Leviton A, Goldstein M, Bax M, Damiano D, et al. A report: the definition and classification of cerebral palsy April 2006. Developmental Medicine & Child Neurology. 2007;49(109):8-14.

16. Carr A, O'Reilly G, Walsh PN, McEvoy J. The Handbook of Intellectual Disability and Clinical Psychology Practice. Hoboken: Routledge; 2014.

17. Demetrious A. Tracing psychology’s invisible giant and its visible guards. In: Sternberg RJ, & Grigorenko, E.L., editor. The General Factor of Intelligence: How general is it? New York: Psychology Press; 2012. p. 3-18.

18. Gottfredson LS. Mainstream science on intelligence: An editorial with 52 signatories, history, and bibliography. 1997. p. 13-23.

For peer review only - http://bmjopen.bmj.com/site/about/guidelines.xhtml

19. Ortiz SO. CHC Theory of Intelligence. In: Goldstein S, Princiotta D, Naglieri JA, editors. Handbook of Intelligence: Evolutionary Theory, Historical Perspective, and Current Concepts. New York, NY: Springer New York; 2015. p. 209-27.

20. Schneider WJ, McGrew KS. The Cattell-Horn-Carroll Theory of Cognitive Abilities. In: Flanagan DP, McDonough EM, editors. Contemporary Intellectual Assessment:Theories, Tests, and Issues. Fourth ed. New York: The Guildford Press; 2018. p. 73-163.

21. Keith TZ, Reynolds MR. Cattell-Horn-Carroll Abilities and Cognitive Tests: What We've Learned from 20 Years of Research. Psychology in the Schools. 2010;47(7):635-50.

22. Blackwell LS, Rodriguez S, Guerra-Carrillo B. Intelligence as a Malleable Construct. In: Goldstein S, Princiotta D, Naglieri JA, editors. Handbook of Intelligence: Evolutionary Theory, Historical Perspective, and Current Concepts. New York, NY: Springer New York; 2015. p. 263-82.

23. Cassidy S, Roche B, Colbert D, Stewart I, Grey IM. A relational frame skills training intervention to increase general intelligence and scholastic aptitude. Learning and Individual Differences. 2016;47:222-35.

24. Au J, Sheehan E, Tsai N, Duncan G, Buschkuehl M, Jaeggi S. Improving fluid intelligence with training on working memory: a meta-analysis. Psychonomic Bulletin & Review. 2015;22(2):366-77.

25. Jaeggi SM, Buschkuehl M, Jonides J, Perrig WJ. Improving fluid intelligence with training on working memory. Proceedings of the National Academy of Sciences. 2008;105(19):6829-33.

26. Hayes TR, Petrov AA, Sederberg PB. Do we really become smarter when our fluid-intelligence test scores improve? Intelligence. 2015;48:1-14.

For peer review only

27. Harrison TL, Shipstead Z, Hicks KL, Hambrick DZ, Redick TS, Engle RW. Working Memory Training May Increase Working Memory Capacity but Not Fluid Intelligence. Psychological Science. 2013;24(12):2409-19.

28. Törneke N. Learning RFT an introduction to relational frame theory and its clinical applications. Ebooks C, ProQuest, editors: Oakland, CA : Context Press; 2010.

29. Rudd L, Lambert M, Satterwhite M, Zaier A. Mathematical Language in Early Childhood Settings: What Really Counts? Early Childhood Education Journal. 2008;36(1):75-80.

30. Wechsler D. WISC-V: Technical and interpretive manual. Bloomington: Pearson; 2014.

31. Wechsler D. Wechsler Intelligence Scale for Children - Fifth Edition Australian and New Zealand Administration and Scoring Manual. Sydney: NCS Pearson; 2016.

32. Joshua N, Wilson C, Bendrups N. WIAT-III A&NZ Examiner's Manual. Sydney: NCS Pearson; 2016.

33. Gioia GA, Isquith PK, Kenworthy L, Barton RM. Profiles of Everyday Executive Function in Acquired and Developmental Disorders. Child Neuropsychology. 2002;8(2):121-37.

34. Goodman R. Psychometric Properties of the Strengths and Difficulties Questionnaire. Journal of the American Academy of Child & Adolescent Psychiatry. 2001;40(11):1337-45.

35. Rutter M, Bailey A, Lord C. The Social Communication Questionnaire. Los Angeles: Western Psychological Services; 2003.

36. Reynolds CR, Kamphaus RW. Behavior Assessment System for Children. Third ed. Bloomington: Pearson; 2015.

37. Conners CK. Conners' Rating Scales - Revised Technical Manual. Toronto: Multi-Health Systems; 1997.

For peer review only - http://bmjopen.bmj.com/site/about/guidelines.xhtml

38. Waters E, Davis E, Mackinnon A, Boyd R, Graham HK, Kai Lo S, et al. Psychometric properties of the quality of life questionnaire for children with CP. Developmental Medicine & Child Neurology. 2007;49(1):49-55.

39. Muris P, Meesters C, Eijkelenboom A, Vincken M. The self-report version of the Strengths and Difficulties Questionnaire: Its psychometric properties in 8- to 13-year-old non-clinical children. British Journal of Clinical Psychology. 2004;43(4):437-48.

40. Wei T, Chesnut S, Barnard-Brak L, Richman D. Psychometric Analysis of the Social Communication Questionnaire Using an Item-Response Theory Framework: Implications for the Use of the Lifetime and Current Forms. Journal of Psychopathology and Behavioral Assessment. 2015;37(3):469-80.

41. Faul F, Erdfelder E, Lang A-G, Buchner A. G*Power 3: A flexible statistical power analysis program for the social, behavioral, and biomedical sciences. Behavior Research Methods. 2007;39(2):175-91.

For peer review only

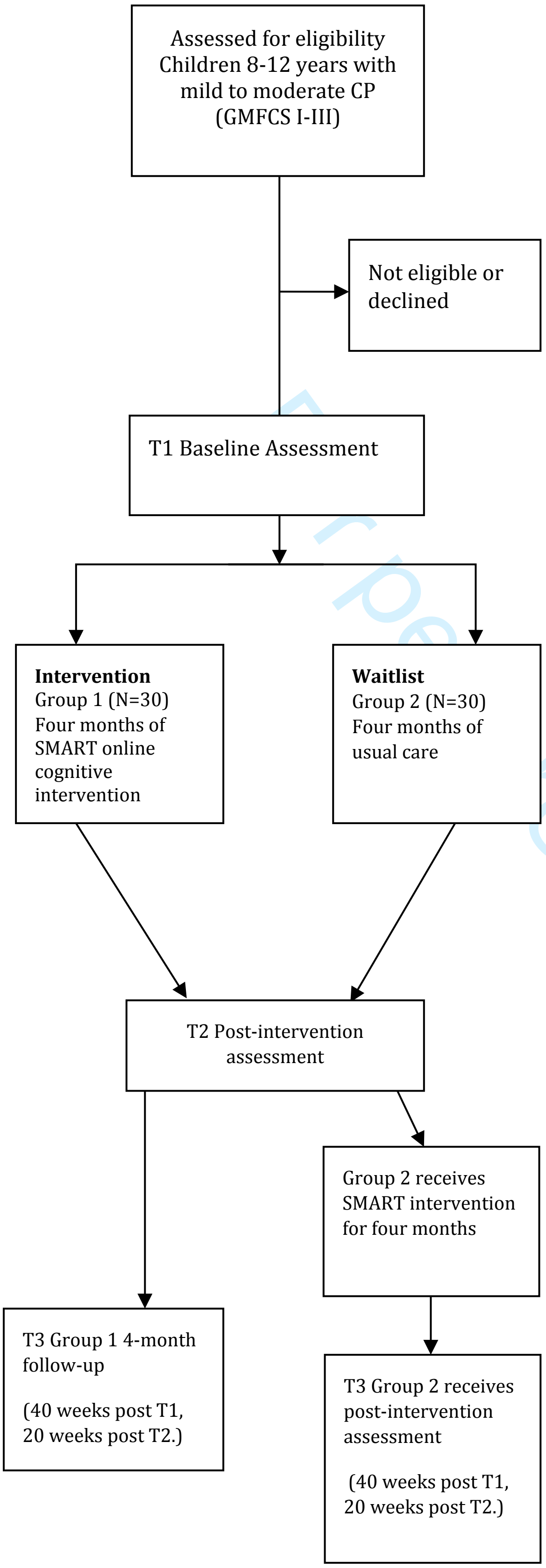

Measures (T1, T2, T3)

Child:
Intellectual Functioning (WISC-V)
Academic Achievement (WIAT-III)
Social-Emotional Functioning (SDQ – Child self-report)

Parent:
Executive Function (BRIEF)
Attention and Behaviour (Conners-3)
Social-Emotional Functioning (BASC-3, SDQ – Parent report)
Social Communication (SCQ –Current)
Quality of Life (CP-QOL Child – Parent report)

Figure 1. CONSORT Flowchart for SMART protocol

For peer review only

For peer review only - http://bmjopen.bmj.com/site/about/guidelines.xhtml

BMJ Open

A randomised controlled trial of a novel online cognitive rehabilitation program for children with cerebral palsy: A study protocol

Journal:	*BMJ Open*
Manuscript ID	bmjopen-2018-028505.R1
Article Type:	Protocol
Date Submitted by the Author:	27-Mar-2019

Complete List of Authors:	Wotherspoon, Jane; The University of Queensland, Queensland Cerebral Palsy and Rehabilitation Research Centre<br>Whittingham, Koa; The University of Queensland, Queensland Cerebral Palsy and Rehabilitation Research Centre<br>Boyd, Roslyn; The University of Queensland, Queensland Cerebral Palsy and Rehabilitation Research Centre<br>Sheffield, Jeanie; The University of Queensland, School of Psychology

**Primary Subject Heading**:	Paediatrics
Secondary Subject Heading:	Rehabilitation medicine
Keywords:	Developmental neurology & neurodisability < PAEDIATRICS, Developmental Psychology, Cognitive Impairment, Cognitive Training

SCHOLARONE™ Manuscripts

A randomised controlled trial of a novel online cognitive rehabilitation program for children with cerebral palsy: A study protocol

Researchers: Ms Jane Wotherspoon[1]

Dr Koa Whittingham[1] (Chief Investigator)

Professor Roslyn N. Boyd, PhD[1,2]

Dr Jeanie Sheffield[2]

Affiliations: [1]Queensland Cerebral Palsy and Rehabilitation Research Centre (QCPRRC), The University of Queensland

[2]Faculty of Medicine, The University of Queensland

[3]School of Psychology, The University of Queensland

Correspondence to Jane Wotherspoon, Queensland Cerebral Palsy and Rehabilitation Research Centre (QCPRRC), 62 Graham Street, South Brisbane, Queensland, 4101. E-mail: j.wotherspoon@uq.edu.au

For peer review only - http://bmjopen.bmj.com/site/about/guidelines.xhtml

Abstract

Introduction

Cerebral palsy (CP) is the most common cause of physical disability in children, with an estimated 600-700 infants born with CP in Australia each year. Cerebral palsy is typically associated with motor impairments, but nearly half of all children with CP also experience cognitive impairment, potentially impacting educational and vocational achievement. This paper reports the protocol for a randomised controlled trial of a computerised cognitive training intervention based on behavioural principles, Strengthening Mental Abilities through Relational Training (SMART). The study aims to investigate SMART's effect on fluid reasoning, executive function and academic achievement in children with CP.

Methods and Analysis

Sixty children with mild to moderate CP (Gross Motor Function Classification Scale – GMFCS I - IV) aged between 8 and 12 will be recruited. Participants will be randomly allocated to two groups: SMART cognitive training and wait list control. Families will access the program at home over a four-month period. Assessments will be administered at baseline, 20 weeks and at 40-week follow-up for retention. The primary outcome will be fluid intelligence, while academic achievement, executive function and social and emotional wellbeing will be secondary outcomes.

Ethics and Dissemination

This study has approval from the Children's Health Queensland Hospital and Health Service Research Ethics Committee (HREC/14/QRCH/377) and The University of Queensland (2017001806). If the computerised cognitive training program is found to be effective, dissemination of these findings would assist children with CP by providing an accessible, cost-effective intervention that can be completed at home at the individual's own pace.

For peer review only - http://bmjopen.bmj.com/site/about/guidelines.xhtml

Registration Details

This trial is registered with the Australian New Zealand Clinical Trials Registry, Trial ID: ACTRN12617001550392. The study was registered prospectively on 10 November 2017 to present. Recruitment is now under way and we aim to complete recruitment by June 2019, with data collection finalised by March 2020.

Strengths and limitations of this study

- This is the first study to trial the effectiveness of a computerised cognitive intervention in maximising fluid intelligence in children with cerebral palsy.
- It is the first randomised controlled trial of a computerised cognitive intervention based on relational frame theory for children with a developmental disability.
- Interventions for cognitive impairment in this population are not readily available, and if effective, this intervention would provide a cost-effective, easily accessible intervention.
- All participants will receive access to the computerised cognitive training intervention prior to the end of the study.
- No active control group is included in this study; therefore we cannot determine impact of the intervention independent of potential placebo or expectancy effects arising from focused use of a computer program.

Introduction

Cerebral palsy (CP), with a prevalence of 1.4 per 1000 live births,[1] is the most common cause of physical disability in children and an estimated 600-700 infants are born with CP in Australia each year.[2] While CP is typically associated with motor impairments, research is now focusing on accompanying cognitive and executive functioning (EF) deficits and how

For peer review only - http://bmjopen.bmj.com/site/about/guidelines.xhtml

these impact daily living.[3] It is estimated that approximately 45% of children with CP will have an intellectual impairment impacting significantly on educational achievement.[4-7] A number of studies have found cognitive impairment in CP is associated with long-term difficulties in completing formal education, obtaining competitive employment and living independently.[8-11]

While there is growing awareness of cognitive and EF limitations, interventions for CP are typically associated with improving physical activity, limb function, and participation in daily living activities.[12] Few interventions target cognitive function and academic abilities.[12, 13] A review looking at new technologies in the treatment of CP and developmental coordination disorder found no studies investigating specific cognitive interventions in these groups. [14] A small number of prior studies have examined whether aspects of cognitive function can be improved, through either web-based multimodal therapy ("Move it to Improve it" [Mitii])[15] or a mindfulness-based yoga programme (MiYoga)[16], A randomised controlled trial of Mitii measured visual perceptual skills of participants and found a significant effect of the web-based therapy program on visual-perceptual skill, although concluded that the effect was not of clinical significance. [15] Mak et al. found a mindfulness-based movement intervention demonstrated significant better sustained attention post-intervention than a waitlist control group, but no differences were found for other measures associated with cognition, including working memory and executive function. [16]

For children with CP who live in remote or isolated communities, access to clinic-based interventions is further limited. This study aims to trial a novel online cognitive rehabilitation program for children with CP targeted to improve intellectual functioning, EF, and educational achievement. The program - Strengthening Mental Abilities Through Relational Training (SMART)[17] - is founded upon relational frame theory, which proposes

For peer review only - http://bmjopen.bmj.com/site/about/guidelines.xhtml

that the development of language and complex reasoning in humans rests upon our ability derive relations between stimuli arbitrarily and without direct experience. An online program designed to train relational framing ability and potentially improve complex reasoning would be a cost-effective, accessible intervention for children with CP.

Cerebral palsy and cognitive impairment

Cerebral palsy refers to a group of motor disorders, originating in a non-progressive injury or disturbance to the brain.[18-20] These disturbances occur early in development, impacting the foetal or infant brain.[19] Cerebral palsy is associated with various types of brain lesions, [21] arising from many different causes, congenital and acquired, including intracranial haemorrhage, asphyxia, prematurity, low birth weight or infection.[22]

While motor impairment is a defining feature of CP, more recent definitions acknowledge the frequent comorbidities, such as vision problems, epilepsy, and cognitive and communication difficulties.[22, 23] Fewer studies have focused on cognitive impairment in cerebral palsy than on motor impairment, but the research that has been undertaken suggests a significant proportion of individuals with CP have impaired cognitive function.[7] A systematic review of rates of impairment in CP found that 49% of children with CP had an intellectual disability, defined as an IQ below 70, while 28% had a severe intellectual disability, with an IQ below 50.[21] Similarly, a population-based study of more than 1,100 individuals with CP in Australia found 45% had been recorded as having an intellectual disability, although level of impairment could not be determined for many in this sample.[7] It is recognised that although CP is a non-progressive disease, the impact of these additional impairments can exert significant influence over a child’s development, impacting academic and vocational outcomes, psychological well-being and quality of life,[21] as well as general health.[7]

For peer review only - http://bmjopen.bmj.com/site/about/guidelines.xhtml

Fluid Intelligence

Amongst most intelligence researchers, there is broad agreement that intelligence is associated with certain abilities, such as problem solving, understanding abstract ideas, or capacity for learning.[24-26] One prominent contemporary model, and the most influential at present in the field of intelligence testing, is the Cattell-Horn-Carroll (CHC) theory of intelligence.[27, 28] CHC theory offers a system for classifying cognitive ability that has allowed greater consensus in the literature around what is being measured and referred to by terms such as crystallised and fluid intelligence.[28] Fluid intelligence is a measure of the capacity to solve novel problems and reason abstractly, while crystallised intelligence is a measure of comprehension and acquired knowledge.[29]

While in the past, intelligence was considered a stable attribute, more recently, it has been conceptualised as a quality open to change and development.[30] As a result, attention has turned to potential factors that could play a role in determining intelligence, with the aim of developing interventions.[31] Furthermore, by combining technological innovation with proposed models of cognitive plasticity, the possibility of accessible interventions, delivered via computers, iPads or similar devices, has emerged.[14] For example, Løhaugen et al.[32] have proposed a randomised controlled trial assessing the efficacy of computer-based working memory training in children with cerebral palsy.

Various online cognitive training programs have been developed and many of these have targeted working memory (WM), an executive function that involves the ability to temporarily hold and manipulate information. Some researchers have hypothesised that gains in WM could transfer into gains in fluid intelligence.[33, 34] Research into this area is ongoing, and results are mixed.[33] While Jaeggi et al.[34] generated great interest in cognitive

For peer review only - http://bmjopen.bmj.com/site/about/guidelines.xhtml

training when they reported gains in fluid intelligence after WM training,[35] numerous studies have failed to replicate such transfer effects,[36] and research continues in this field.

SMART program

The novel online cognitive training program to be trialled, Strengthening Mental Abilities through Relational Training (SMART), shows promise in pilot studies in helping improve children's cognitive skills (e.g., ability to learn, think, and reason).[17] SMART is a web-based cognitive training program, currently available in English or Dutch, that directly trains the relational abilities thought to be foundational to complex cognition. SMART is grounded in contextual behavioural science, specifically Relational Frame Theory,[17] which proposes that all human language and complex cognition is underpinned by our ability to relate stimuli arbitrarily**.** That is, humans can relate stimuli in a manner that does not correspond to the physical properties of the stimuli. Such relations between stimuli are called relational frames, and a number of such frames exist, such as coordination (e.g., same as), comparative (e.g., more/less), and temporal frames (e.g., before and after).[37] An example of a relational frame of more/less would be with Australian one- and two-dollar coins, where individuals learn that the two-dollar coin is worth more, even though it is physically smaller than the one-dollar coin.

Many relational frames, including spatial (e.g. under), measurement (e.g. bigger) and ordinal (e.g. first) are foundational to mathematics.[38] Repeated exposure to spatial, measurement, ordinal and other mathematics-relevant relational frames is considered a critical part of early education, helping students develop their understanding of mathematical concepts.[38]

In addition to learning relational frames, people can also learn behavioural responses through derived relations, not merely through direct experience.[17] Through derived relations, our relational framing abilities greatly enhance our capacity to learn and to interact effectively with others and our environment. As an example, a person might learn that the star Vega is

For peer review only - http://bmjopen.bmj.com/site/about/guidelines.xhtml

closer to Earth than Canopus, but further than Sirius. They could then derive that Canopus is the furthest star from Earth of the three, even though it has not been directly taught, and Canopus appears brighter than Vega. As relational framing is foundational to complex cognition, the direct training of relational framing itself has the potential to have wide-reaching effects on cognition.

The SMART program itself consists of 55 modules that can be worked through at the participant's own pace. Progress to each module requires successful completion of the preceding one. A maximum of five modules can be completed per day. Each module presents a proposition in the form of a relations between nonsense words, and then asks a yes/no question based on the proposition. For example, "SAJ is the same as MIS. Is MIS the same as SAJ?". Derived relations are also trained through the addition of more than two nonsense words. For example, "SAJ is the same as MIS. QUW is the same as SAJ." Is QUW the same as MIS?" Each module provides multiple examples of the relationship being trained, and if 16 questions are answered correctly, each within a 30-second time frame, the next module is unlocked. Additional relations trained include *opposite*, *more than* and *less than*.

A feasibility pilot study of SMART was conducted over a 9-month period with eight children aged 11-12 years old who had been experiencing educational difficulties at school.[17] Seven of the eight children showed significant increases in their intellectual functioning (as measured by the Wechsler Intelligence Scale for Children – Fourth Edition), an improvement of more than one standard deviation. A 2016 study found similar significant increases in IQ, with a sample size of 15 11- to 12-year-old children. [31] While promising, practice effects need to be accounted for when repeated administration of standardised measures of intelligence occurs, as they may influence performance, with average gains of 6-7 points over a one-month period found for the WISC-V measure of full-scale IQ [39]. Furthermore, assessment of fluid reasoning ability may be more affected by practice effects than verbal or working memory

For peer review only - http://bmjopen.bmj.com/site/about/guidelines.xhtml

tasks [39], as fluid reasoning tasks are associated with ability to solve novel problems. A randomised controlled design rather than pre- and post-intervention studies could control for practice effects, but the two studies that have utilised this design have been limited by small sample sizes and high attrition rates. [40, 41] To date, no studies have investigated the efficacy of SMART in the cerebral palsy population.

The underlying cognitive skills trained in the program are required for vocabulary acquisition, mathematical reasoning, and other academic and learning skills. The program can be accessed in the client's home at their convenience via iPad, Mac or PC with Internet access and can be completed at the child's own pace. As such, it is a potentially cost-effective solution that can be delivered to children with CP who are unable to access ongoing rehabilitation services.

METHODS

Specific Aims

The aim of this randomised controlled trial is to test the efficacy of a novel web-based cognitive rehabilitation program for children aged between 8 and 12 years old with mild to moderate congenital CP.

Hypotheses

The primary hypothesis to be tested is that in a randomised controlled trial for children aged 8-12 with CP:

1. Participants in the intervention group will demonstrate improved on a standardised test of intellectual ability performance immediately post-intervention when compared to a waitlist control group receiving care as usual. (Wechsler Intelligence Scale for Children - Fifth Edition; WISC-V)[42, 43]

The secondary hypotheses to be tested are that the SMART intervention group will demonstrate improvements in the following outcomes:

For peer review only - http://bmjopen.bmj.com/site/about/guidelines.xhtml

1. Academic achievement (Wechsler Individual Achievement Test Third Edition; WIAT-III)[44]

2. Executive function (Behavior Rating Inventory of Executive Function; BRIEF)[45]

3. Social and emotional functioning (Strengths and Difficulties Questionnaire; SDQ; Behavior Assessment System for Children - Third Edition; BASC-3; Social Communication Questionnaire; SCQ;)[46-48].

4. Attention (Conners - Third edition; Conners-3)[49].

5. Quality of life (Cerebral Palsy Quality of Life - Child; CP-QOL)[50].

Study Design

The study is a randomised controlled trial design with waitlist control group, to determine the effectiveness of the SMART program for children with CP aged 8-12 years (see CONSORT flowchart in Figure 1.) After baseline assessment, participants will be randomly allocated to either the intervention or waitlist control group.

Outcomes will be assessed for all participants at three timepoints, baseline, at 20 weeks post-baseline, and at 40 weeks post-baseline. The intervention group will commence the SMART program immediately and complete it over the following 20 weeks, before undergoing post-intervention assessment at 20 weeks post-baseline and follow-up assessment at 40 weeks post-baseline. Participants will be provided with log-in details enabling them to access to the online program at no cost via the program website (http://raiseyouriq.com/) for up to five months. They will be able to access it at home via either iPad or computer. The waitlist control group will continue care as usual for 20 weeks before returning for a second assessment visit, at which point they will commence the SMART program. Final assessment for the waitlist control group will be post-intervention at 40 weeks.

Participants: We aim to recruit 60 children with mild to moderate CP (Gross Motor Function Classification Scale – GMFCS I - IV) aged between 8 and 12 years old. All children are

For peer review only - http://bmjopen.bmj.com/site/about/guidelines.xhtml

required to have sufficient cooperation and cognitive understanding to perform the tasks and access the novel online cognitive training program, have access to the internet at home, and be able to attend three assessment sessions in Brisbane. Sufficient cooperation and cognitive understanding will be confirmed at baseline assessment, as participants who are able to undertake the iPad-based assessments will be deemed to meet criteria. Children will be excluded if they have unstable epilepsy (i.e., not controlled by medication); an unstable brain injury (e.g., degenerative or metabolic condition); and/or active medical condition (e.g., chemotherapy, radiotherapy, or neurosurgical).

Recruitment: Participants will be recruited from a consent-based research database at the Queensland Cerebral Palsy and Rehabilitation Research Centre, and through the Queensland Paediatric Rehabilitation Service at the Queensland Children's Hospital. Participants will be enrolled in the study by the first author.

Patient and public involvement

Participants, families and the public were not involved in the design or recruitment of this study. Participants are informed of the study burden from the time of initial contact, and are advised of their ability to withdraw from the study at any time. All participants will receive feedback on the results of the assessments administered once they have completed their involvement in the study.

Randomisation

Baseline assessments and demographic questionnaires will be completed prior to randomisation. Once complete, participants will be randomised to either waitlist control or intervention group. Randomisation will be via stratified random blocks, using a computer-generated block randomisation sequence, stored by staff members unconnected with the study. Allocation to either waitlist control or intervention will be recorded on pieces of paper, and these will be folded, then placed inside opaque envelopes by a staff member not involved

For peer review only - http://bmjopen.bmj.com/site/about/guidelines.xhtml

in the study. Envelopes will be sealed, and only opened upon completion of baseline assessment. Participants will be stratified according to IQ (<70 or ≥70), as measured on baseline assessment.

Blinding

Given the nature of the intervention, participants will not be blinded as to which group they are assigned to. As assessment will be undertaken by the first author as part of a PhD project, assessors will not be blinded in this project.

Adverse events

No health or safety risks associated with participation in this study have been identified, and the risk of adverse events is considered low. Any events associated with either intervention or wait-list control groups over the course of the study will be recorded. If any adverse events are identified, they will be reviewed by the investigators. As the ethical review process and trial conduct is overseen by two ethics committees, no additional data monitoring is considered necessary.

Data Management

Participants will be allocated randomly generated identification codes, and these will be used to de-identify hardcopy and electronic files. Paper copies relating to assessment will be de-identified and physically stored in a locked filing cabinet at the Queensland Cerebral Palsy and Rehabilitation Research Centre. Electronic data will be stored on REDCap, a secure web platform for creating and managing online databases. The installation of REDCap used for this project is hosted bv the University of Queensland and managed by the Queensland Clinical Trials and Biostatistics Centre.

Measures:

For peer review only - http://bmjopen.bmj.com/site/about/guidelines.xhtml

Demographic information will be obtained via a parent survey, gathering information on the participant's background, including gestational age, comorbid diagnoses, and GMFCS classification. Further demographic information includes school year, type of school, and whether any additional teaching support is accessed, along with parent education and household income.

All children will undergo a comprehensive cognitive, psychoeducational, and psychosocial assessment by a psychologist at baseline (i.e., before treatment), and re-assessment at 20 weeks (after intervention for immediate group), and 40 weeks (retention for immediate group, and after intervention for control group). It is noted that many of these assessments have not been validated for children with cerebral palsy, and have been chosen as no valid alternatives are available. However a review of assessments for children with cerebral palsy found that motor involvement, communication and visual impairment were key factors in determining suitability of assessments. [51] We have specifically chosen an iPad-based assessment delivery format for our primary outcome (full-scale IQ), that is similar in motor and language demand to the intervention program itself. If children are able to meet the inclusion criteria for the study, it is anticipated that they will also be able to complete the assessments.

Children will complete the following assessments:

- *The Wechsler Intelligence Scale for Children Fifth Edition (WISC-V)* is a standardised measure of overall cognitive/intellectual functioning. The WISC-V produces a full-scale intelligence IQ score (FSIQ), along with five primary index scores – Verbal Comprehension Index (VCI), Visual-Spatial Index (VSI), Fluid Reasoning Index (FRI), Working Memory Index (WMI), and Processing Speed Index (PSI), each derived from two subtests. The WISC-V has been found to have good internal consistency (α=.96 for FSIQ and α= .86-.94 for primary index scores).[43] Interrater

For peer review only - http://bmjopen.bmj.com/site/about/guidelines.xhtml

reliability has also been found to be acceptable (*r*= .98-.99 for interscorer agreement on a subset of subtests).[42]

- *The Wechsler Individual Achievement Test Third Edition (WIAT-III)* is a standardised measure of educational achievement. It provides measures of achievement across domains of oral language, reading, written language and mathematics. The internal consistency coefficients are good (α=.83-.95), with the exception of a single subtests (Alphabet Writing Fluency, α=.69), which is not used with the age range participating in this study.[44]
- *The Strengths and Difficulties Questionnaire (SDQ) - Child self-report*. This questionnaire measures the child's behaviour and adjustment. The SDQ is a 25-item measure with frequency of behaviour rated on a 3-point Likert scale.[46] The SDQ produces five subscales: emotional symptoms, conduct problems, inattention/hyperactivity, peer problems and prosocial behaviour (range 0-10), and a total difficulties score (range 0-40). Research suggest that for younger children, internal consistency is acceptable for the total difficulties, emotional symptoms, prosocial behaviour, and inattention/hyperactivity subscales, but not for peer and conduct problems.[52] However, Muris et al.[52] note that the scale can provide useful information about psychopathology in children from eight years old.

Parents will also be administered questionnaires during the three assessments points. These include:

- *The Behaviour Rating Inventory of Executive Function (BRIEF)* assesses the child's executive functioning in everyday life. The *BRIEF* is an 85-item parent-rated questionnaire assessing behavioural manifestations of executive functions in everyday life.[45] The *BRIEF* produces a Behavioural Regulation Index (BRI; Initiate, Working

For peer review only - http://bmjopen.bmj.com/site/about/guidelines.xhtml

Memory, Plan/Organise, Organisation of Materials, and Monitor subscales) a Metacognition Index (MCI; Inhibit, Emotional Control, and Shift subscales). The *BRIEF* has been shown to be a valid measure of executive functioning and has good internal consistency (α=.80-.98) and high test-retest reliability on the BRI (r=.92), MCI (r=.88), and the GEC (r=.86).

- *Conners-3 Parent Report,* which assesses childhood behaviours and behavioural disorders including Attention Deficit Hyperactive Disorder in children and adolescents aged 6-18.[49] The Conners-3 consists of 110 statements and takes approximately 20 minutes to complete. The Conners-3 measures seven key areas – inattention, learning problems, aggression, family relations, hyperactivity/impulsivity, executive functioning, and peer relations. Both internal consistency coefficients (α=.83-.94) and test-retest reliability (r=.52-.94) are good.
- The *Behavior Assessment System for Children – 3rd Edition (BASC-3)* measures children's adaptive and problem behaviours and emotional difficulties at home and in community settings [48]. It is completed by a parent or guardian and consists of 175 statements, taking approximately 20 minutes. The *BASC-3* produces five composite scales, Internalizing Problems, Externalizing Problems, School Problems, Adaptive Skills and the Behavioural Symptoms Index. Internal consistency coefficients for the Parent Rating Scale (α=.79-.97 for 6- to 7-year-olds; α=.79-.96 for 8- to 11-year-olds) and test-retest reliability (r=.80-.92 for Child scales) are good.
- *Social Communication Questionnaire – Current, (SCQ - Current)* screens for communication skills and social functioning difficulties associated with autism spectrum disorders. The *SCQ – Current* evaluates communication skills and social functioning in children, based on the previous three months. While research suggests the SCQ – Current has lower internal consistency than the Lifetime version,[53]

For peer review only - http://bmjopen.bmj.com/site/about/guidelines.xhtml

concerns around its use particularly focus on children below five years of age, which is outside the age range of participants in this study, and on using the Current form as an alternative to the Lifetime form for screening for Autism Spectrum Disorder. For the purpose of measuring change over time, however, the Current form is recommended.[47] It contains 40 items and is completed by a parent or primary caregiver in under 10 minutes. Scores on the SCQ provide information on the following domains: Social Interaction, Communication and Stereotyped, Repetitive or Restricted Behaviour.

- *Strengths and Difficulties Questionnaire (SDQ) – Parent Report* measures pro-social, internalising and externalising behaviours in children. It is a 25-item measure with frequency of behaviour rated on a 3-point Likert scale. The *SDQ* is a 25-item parent-report measure of child behaviour and adjustment, with frequency of behaviour rated on a 3-point Likert scale.[46] The SDQ produces five subscales: emotional symptoms, conduct problems, inattention/hyperactivity, peer problems and prosocial behaviour (range 0-10). It produces a total difficulties score (range 0-40) found to have adequate internal reliability (α=.73) and test-retest reliability (*r*=.62) as well as discriminant and concurrent validity.[46]
- *Cerebral Palsy Quality of Life for Children- Parent Report (CP-QOL-Child)* measures child quality of life. *CP QOL-Child* is a 66-item parent-report measure of child quality of life specifically developed for use in children with CP.[50] It measures quality of life across domains including physical wellbeing, social wellbeing, emotional wellbeing, school, service access and social acceptance. It has good concurrent validity, internal consistency (α=.76-.89) and test-retest reliability (*r*=.80-.90).[50]

For peer review only - http://bmjopen.bmj.com/site/about/guidelines.xhtml

The SMART program provides an internal measure of Relational Ability, and all children will complete this measure at baseline, 20 weeks and 40 weeks. In addition, information will be recorded on time taken for each participant to complete the program. Assessments at baseline, 20 and 40 weeks will take approximately two to three hours per session. The waitlist control design ensures all children in the study will receive the intervention within six months of being randomised to either commence the program immediately or after 20 weeks. Table 1 summarises the measures for each assessment point.

**Measures** (T1, T2, T3)
**Child:**<br>Intellectual Functioning (WISC-V)<br>Academic Achievement (WIAT-III)<br>Social-Emotional Functioning (SDQ – Child self-report)<br>Relational Ability (RAI)<br><br>**Parent:**<br>Executive Function (BRIEF)<br>Attention and Behaviour (Conners-3)<br>Social-Emotional Functioning (BASC-3, SDQ – Parent report)<br>Social Communication (SCQ –Current)<br>Quality of Life (CP-QOL Child – Parent report)

Table 1. Summary of Assessment Measures.

Qualitative Interview

At the conclusion of the study, semi-structured interviews will be conducted with children and caregivers by the first author, a registered psychologist. The aim of the interview is to explore participants' engagement with the online cognitive rehabilitation program and gain qualitative insights into families' experience with the program. Questions will cover what families like and disliked about the program, how easy they found it was to access at home, and to remain engaged. If the program is found to be effective, such qualitative insights will

For peer review only - http://bmjopen.bmj.com/site/about/guidelines.xhtml

be valuable in the translation phase. The script for the interview can be found in Supplementary Appendix A. Interviews will be recorded, transcribed and analysed.

Intervention: With the SMART online platform, participants answer problems directly training relational framing and receive immediate feedback on their answers. Study participants are provided with an alias login name (to maintain confidentiality) and a password. Children are encouraged to complete the SMART intervention for 30 minutes per session, for a total of 1.5 hours per week. SMART is incremented and can be completed at the child's own pace. The full dosage is reached when the child completes the entire intervention, which is expected to occur within 20 weeks. Throughout the RCT, a psychologist will regularly contact the family and monitor that child's progress, supporting both parent and child in meeting their goals for SMART, and maintaining engagement.

A resource guide prepared for this study will be provided to all families. This guide provides technical information on accessing the program, a description of how to work through each stage of the program, and information on how parents can support their child to work on the program, including a visual chart to keep track of progress.

Statistical Analysis: Study hypotheses will be analysed by means of appropriate statistical tests, with statistical significance for all tests set at $p<0.05$ with adjustment for multiple comparisons, and all analyses will be intention to treat. We propose to carry out a Benjamini-Hochberg procedure to control for false discoveries.[54]

Mixed analysis of covariance analyses will be conducted with time (baseline and 20 weeks) as the within subjects variable, and group (intervention or waitlist) as the between subjects variable, and baseline data as the covariate. Secondary analysis will profile cognitive change over time for participants based on their test scores. This will include *t* tests and linear

For peer review only - http://bmjopen.bmj.com/site/about/guidelines.xhtml

regression to explore within-subject differences from post-intervention to follow-up, and over three timepoints (baseline, post-intervention and follow-up) for participants in the intervention group.

Power Analysis and Sample Size:

Power analysis was conducted using the software package G*Power [55], for an ANCOVA repeated measures, between factors test. With a sample size of 60 subjects, and assuming an error rate of 5% and p=0.70 for within-subject correlations, this sample size results in 81.37% power to detect a large (Cohen's f=0.40) mean difference of 12 IQ points between groups, after allowing for an attrition rate of 10%.

Ethics and Dissemination

This study has received full ethical approval from the Children's Health Queensland Hospital and Health Service Research Ethics Committee (HREC/14/QRCH/377) and The University of Queensland (2017001806). Protocol modifications and amendments will be submitted to the ethics committees for approval. Amendments to the protocol will be included in publications reporting on outcomes of this study. All families will be provided with a written informed consent form by the first author at the initial visit (Supplementary Appendix B) that they will be required to sign before commencing participation. This trial has been registered with the Australian New Zealand Clinical Trials Registry, Trial ID: ACTRN12617001550392. Study results will be disseminated through publication in scientific journals and participation in conferences. The authors of this protocol will be authors of any publications describing study outcomes, and professional writers will not be used. Families who participate in the study will receive information on the study results, as well as a feedback report on the outcomes of assessments their child has completed. If the computerised cognitive training program is found to be effective, dissemination of these

For peer review only - http://bmjopen.bmj.com/site/about/guidelines.xhtml

findings would assist children with CP by providing an easily accessible, cost-effective intervention that can be completed at home at the individual's own pace.

Trial Progress

This protocol is Version 2, 16 March 2019. The study is currently actively recruiting participants, after initial recruitment commenced in June 2018.

Discussion

This protocol paper has reported the background and study design for a randomised controlled trial investigating the effectiveness of a computerised cognitive training program for children with CP. This program has not previously been studied in this population. The research study will assess children's cognitive skills, executive ability and social and emotional functioning, with fluid intelligence the primary outcome of interest. Qualitative information will be gathered on families' experience engaging with the program. Results of the study will be disseminated through peer-reviewed journals and at relevant scientific conferences.

Nearly half of all children with CP are estimated to also have an intellectual impairment, impacting academic achievement and ability to achieve educational and vocational goals in the long-term. If this computerised cognitive training program is found to be effective, a flexible, easily accessible intervention will be available for this population, where at present there at few options available for addressing difficulties with cognitive skills in CP.

Contributors

The study was designed and established by all the authors. JW is responsible for the ethics application and reporting. JW is responsible for recruitment and data collection. JW will take a lead role in preparing publications on the clinical outcomes of the study. KW, RB and JS

For peer review only - http://bmjopen.bmj.com/site/about/guidelines.xhtml

will contribute to the preparation of publications and are providing supervision throughout the study. JW will take on a lead role for statistical analysis. JW drafted the final version of this manuscript, while all authors critically reviewed and approved the final version. JW will use data from this study to contribute to her PhD thesis.

Funding

JW is a PhD scholar funded by an Australian Government Research Training Program Stipend and Queensland Cerebral Palsy and Research Rehabilitation Centre Top-up Scholarship.

Competing Interests

The authors state they have no competing interests in this project.

For peer review only

References

1. Group ACPR. Report of the Australian Cerebral Palsy Register Birth years 1995-2012, November 2018 2018.

2. Economics A. The economic impact of cerebral palsy in Australia in 2007. Canberra: Access Economics; 2008.

3. Bottcher L, Flachs EM, Uldall P. Attentional and Executive Impairments in Children with Spastic Cerebral Palsy. Developmental Medicine & Child Neurology. 2010;52(2):e42-e7.

4. Himmelmann K, Beckung E, Hagberg G, Uvebrant P. Gross and fine motor function and accompanying impairments in cerebral palsy. Developmental Medicine & Child Neurology. 2006;48(6):417-23.

5. Rankin J, Cans C, Garne E, Colver A, Dolk H, Uldall P, et al. Congenital anomalies in children with cerebral palsy: a population-based record linkage study. Developmental Medicine & Child Neurology. 2010;52(4):345-51.

6. Andersen GL, Irgens LM, Haagaas I, Skranes JS, Meberg AE, Vik T. Cerebral palsy in Norway: Prevalence, subtypes and severity. European Journal of Paediatric Neurology. 2008;12(1):4-13.

7. Reid SM, Meehan EM, Arnup SJ, Reddihough DS. Intellectual disability in cerebral palsy: a population-based retrospective study. Developmental Medicine & Child Neurology. 2018;60(7):687-94.

8. Benner JL, Hilberink SR, Veenis T, van der Slot WMA, Roebroeck ME. Course of employment in adults with cerebral palsy over a 14-year period. Developmental Medicine & Child Neurology. 2017;59(7):762-8.

For peer review only - http://bmjopen.bmj.com/site/about/guidelines.xhtml

9. Mesterman R, Leitner Y, Yifat R, Gilutz G, Levi-Hakeini O, Bitchonsky O, et al. Cerebral Palsy—Long-Term Medical, Functional, Educational, and Psychosocial Outcomes. Journal of Child Neurology. 2009;25(1):36-42.

10. Michelsen SI, Uldall P, Kejs AMT, Madsen M. Education and employment prospects in cerebral palsy. Developmental Medicine & Child Neurology. 2005;47(8):511-7.

11. Murphy KP, Molnar GE, Lankasky K. Employment and social issues in adults with cerebral palsy. Archives of Physical Medicine and Rehabilitation. 2000;81(6):807-11.

12. Novak I, McIntyre S, Morgan C, Campbell L, Dark L, Morton N, et al. A systematic review of interventions for children with cerebral palsy: state of the evidence. Developmental Medicine & Child Neurology. 2013;55(10):885-910.

13. Løhaugen GCC, Beneventi H, Andersen GL, Sundberg C, Østgård HF, Bakkan E, et al. Do children with cerebral palsy benefit from computerized working memory training? Study protocol for a randomized controlled trial. Trials. 2014;15(1):269.

14. Wilson P, Green D, Caeyenberghs K, Steenbergen B, Duckworth J. Integrating New Technologies into the Treatment of CP and DCD. Current Developmental Disorders Reports. 2016;3(2):138-51.

15. James S, Ziviani J, Ware RS, Boyd RN. Randomized controlled trial of web-based multimodal therapy for unilateral cerebral palsy to improve occupational performance. Developmental Medicine & Child Neurology. 2015;57(6):530-8.

16. Mak C, Whittingham K, Cunnington R, Boyd RN. Effect of mindfulness yoga programme MiYoga on attention, behaviour, and physical outcomes in cerebral palsy: a randomized controlled trial. Developmental Medicine & Child Neurology. 2018;60(9):922-32.

17. Cassidy S, Roche B, Hayes SC. A relational frame training intervention to raise intelligence quotients: A pilot study. The Psychological Record. 2011;61(2):173-98.

For peer review only

18. Badawi N, Watson L, Petterson B, Blair E, Slee J, Haan E, et al. What constitutes cerebral palsy? Developmental Medicine & Child Neurology. 1998;40(8):520-7.

19. Rosenbaum P. The Definition and Classification of Cerebral Palsy: Are We Any Further Ahead in 2006? NeoReviews. 2006;7(11):e569-e74.

20. Smithers-Sheedy H, Badawi N, Blair E, Cans C, Himmelmann K, Krägeloh-Mann I, et al. What constitutes cerebral palsy in the twenty-first century? Developmental Medicine & Child Neurology. 2014;56(4):323-8.

21. Novak I, Hines M, Goldsmith S, Barclay R. Clinical prognostic messages from a systematic review on cerebral palsy. Pediatrics. 2012;130(5):e1285.

22. Straub K, Obrzut J. Effects of Cerebral Palsy on Neuropsychological Function. Journal of Developmental and Physical Disabilities. 2009;21(2):153-67.

23. Rosenbaum P, Paneth N, Leviton A, Goldstein M, Bax M, Damiano D, et al. A report: the definition and classification of cerebral palsy April 2006. Developmental Medicine & Child Neurology. 2007;49(109):8-14.

24. Carr A, O'Reilly G, Walsh PN, McEvoy J. The Handbook of Intellectual Disability and Clinical Psychology Practice. Hoboken: Routledge; 2014.

25. Demetriou A. Tracing psychology's invisible giant and its visible guards. In: Sternberg RJ, & Grigorenko, E.L., editor. The General Factor of Intelligence: How general is it? New York: Psychology Press; 2012. p. 3-18.

26. Gottfredson LS. Mainstream science on intelligence: An editorial with 52 signatories, history, and bibliography. 1997. p. 13-23.

27. Ortiz SO. CHC Theory of Intelligence. In: Goldstein S, Princiotta D, Naglieri JA, editors. Handbook of Intelligence: Evolutionary Theory, Historical Perspective, and Current Concepts. New York, NY: Springer New York; 2015. p. 209-27.

For peer review only

28. Schneider WJ, McGrew KS. The Cattell-Horn-Carroll Theory of Cognitive Abilities. In: Flanagan DP, McDonough EM, editors. Contemporary Intellectual Assessment:Theories, Tests, and Issues. Fourth ed. New York: The Guildford Press; 2018. p. 73-163.

29. Keith TZ, Reynolds MR. Cattell-Horn-Carroll Abilities and Cognitive Tests: What We've Learned from 20 Years of Research. Psychology in the Schools. 2010;47(7):635-50.

30. Blackwell LS, Rodriguez S, Guerra-Carrillo B. Intelligence as a Malleable Construct. In: Goldstein S, Princiotta D, Naglieri JA, editors. Handbook of Intelligence: Evolutionary Theory, Historical Perspective, and Current Concepts. New York, NY: Springer New York; 2015. p. 263-82.

31. Cassidy S, Roche B, Colbert D, Stewart I, Grey IM. A relational frame skills training intervention to increase general intelligence and scholastic aptitude. Learning and Individual Differences. 2016;47:222-35.

32. Løhaugen GCC, Beneventi H, Andersen GL, Sundberg C, Ostgard HF, Bakkan E, et al. Do children with cerebral palsy benefit from computerized working memory training? Study protocol for a randomized controlled trial. Trials. 2014;15(1).

33. Au J, Sheehan E, Tsai N, Duncan G, Buschkuehl M, Jaeggi S. Improving fluid intelligence with training on working memory: a meta-analysis. Psychonomic Bulletin & Review. 2015;22(2):366-77.

34. Jaeggi SM, Buschkuehl M, Jonides J, Perrig WJ. Improving fluid intelligence with training on working memory. Proceedings of the National Academy of Sciences. 2008;105(19):6829-33.

35. Hayes TR, Petrov AA, Sederberg PB. Do we really become smarter when our fluid-intelligence test scores improve? Intelligence. 2015;48:1-14.

For peer review only - http://bmjopen.bmj.com/site/about/guidelines.xhtml

36. Harrison TL, Shipstead Z, Hicks KL, Hambrick DZ, Redick TS, Engle RW. Working Memory Training May Increase Working Memory Capacity but Not Fluid Intelligence. Psychological Science. 2013;24(12):2409-19.

37. Törneke N. Learning RFT : an introduction to relational frame theory and its clinical applications

Learning RFT an introduction to relational frame theory and its clinical applications. Ebooks C, ProQuest, editors: Oakland, CA : Context Press; 2010.

38. Rudd L, Lambert M, Satterwhite M, Zaier A. Mathematical Language in Early Childhood Settings: What Really Counts? Early Childhood Education Journal. 2008;36(1):75-80.

39. Kaufman AS, Raiford SE, Coalson DL. Intelligent testing with the WISC-V. Hoboken, NJ, US: John Wiley & Sons Inc; 2016. xviii, 814-xviii, p.

40. Thirus J, Starbrink M, Jansson B. Relational frame theory, mathematical and logical skills: A multiple exemplar training intervention to enhance intellectual performance. International Journal Of Psychology &Amp. 2016;16(2):141-55.

41. Colbert D, Tyndall I, Roche B, Cassidy SJJoBE. Can SMART Training Really Increase Intelligence? A Replication Study. 2018;27(4):509-31.

42. Wechsler D. WISC-V: Technical and interpretive manual. Bloomington: Pearson; 2014.

43. Wechsler D. Wechsler Intelligence Scale for Children - Fifth Edition Australian and New Zealand Administration and Scoring Manual. Sydney: NCS Pearson; 2016.

44. Joshua N, Wilson C, Bendrups N. WIAT-III A&NZ Examiner's Manual. Sydney: NCS Pearson; 2016.

For peer review only - http://bmjopen.bmj.com/site/about/guidelines.xhtml

45. Gioia GA, Isquith PK, Kenworthy L, Barton RM. Profiles of Everyday Executive Function in Acquired and Developmental Disorders. Child Neuropsychology. 2002;8(2):121-37.

46. Goodman R. Psychometric Properties of the Strengths and Difficulties Questionnaire. Journal of the American Academy of Child & Adolescent Psychiatry. 2001;40(11):1337-45.

47. Rutter M, Bailey A, Lord C. The Social Communication Questionnaire. Los Angeles: Western Psychological Services; 2003.

48. Reynolds CR, Kamphaus RW. Behavior Assessment System for Children. Third ed. Bloomington: Pearson; 2015.

49. Conners CK. Conners' Rating Scales - Revised Technical Manual. Toronto: Multi-Health Systems; 1997.

50. Waters E, Davis E, Mackinnon A, Boyd R, Graham HK, Kai Lo S, et al. Psychometric properties of the quality of life questionnaire for children with CP. Developmental Medicine & Child Neurology. 2007;49(1):49-55.

51. Yin Foo R, Guppy M, Johnston LM. Intelligence assessments for children with cerebral palsy: a systematic review. Developmental medicine and child neurology. 2013;55(10):911-8.

52. Muris P, Meesters C, Eijkelenboom A, Vincken M. The self-report version of the Strengths and Difficulties Questionnaire: Its psychometric properties in 8- to 13-year-old non-clinical children. British Journal of Clinical Psychology. 2004;43(4):437-48.

53. Wei T, Chesnut S, Barnard-Brak L, Richman D. Psychometric Analysis of the Social Communication Questionnaire Using an Item-Response Theory Framework: Implications for the Use of the Lifetime and Current Forms. Journal of Psychopathology and Behavioral Assessment. 2015;37(3):469-80.

For peer review only

For peer review only - http://bmjopen.bmj.com/site/about/guidelines.xhtml

54. Benjamini Y, Hochberg Y. Controlling the False Discovery Rate: A Practical and Powerful Approach to Multiple Testing. Journal of the Royal Statistical Society Series B (Methodological). 1995;57(1):289-300.

55. Faul F, Erdfelder E, Lang A-G, Buchner A. G*Power 3: A flexible statistical power analysis program for the social, behavioral, and biomedical sciences. Behavior Research Methods. 2007;39(2):175-91.

For peer review only

For peer review only - http://bmjopen.bmj.com/site/about/guidelines.xhtml

Figure Legends

Figure 1 - Flow Chart of SMART trial design.

For peer review only

For peer review only - http://bmjopen.bmj.com/site/about/guidelines.xhtml

Flow Chart of Study Design

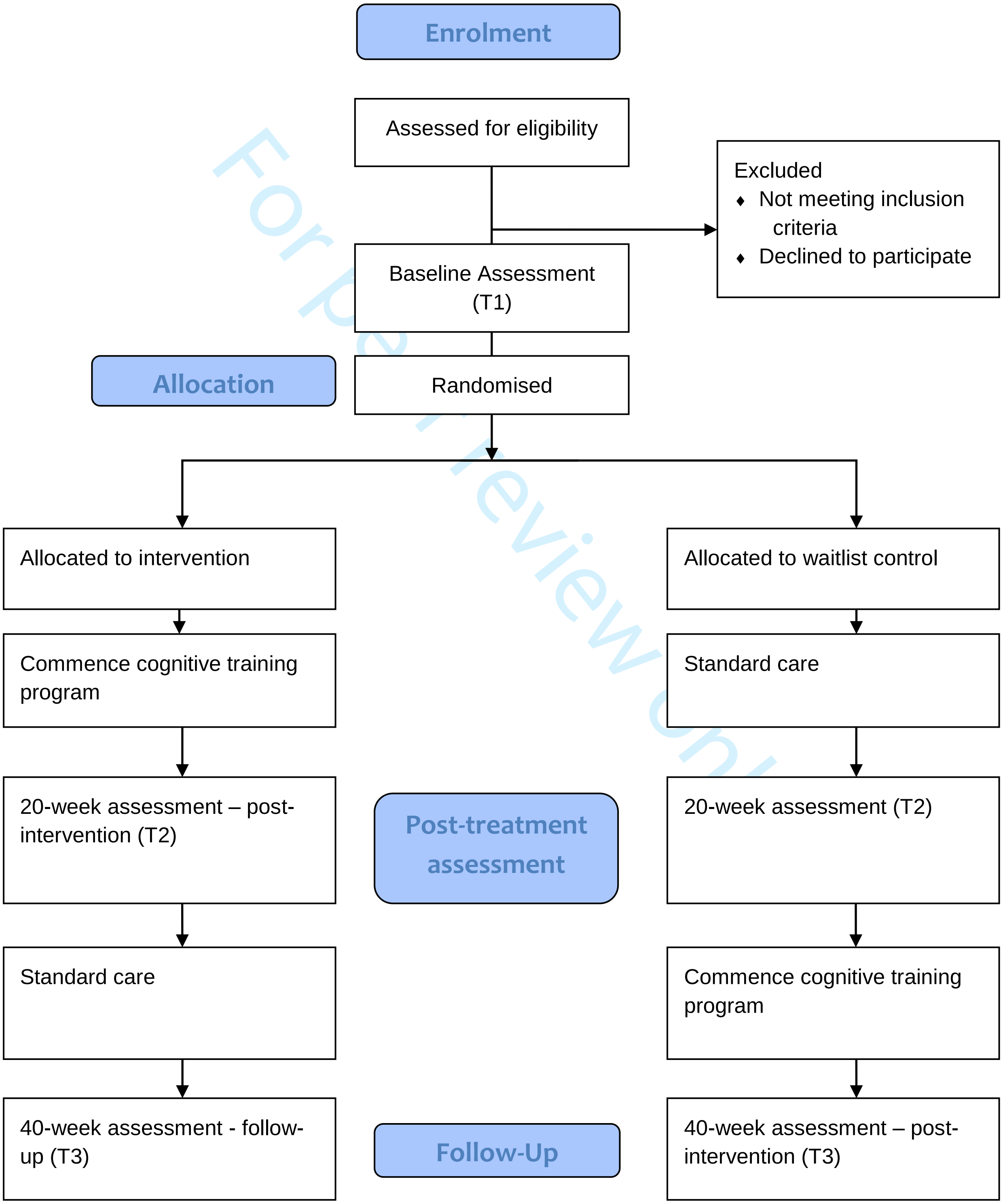

Appendix A

SMART Study Qualitative Interview Questions.

Thank you for taking part in the SMART study. This study has been conducted as part of PhD project through the Queensland Cerebral Palsy and Rehabilitation Research Centre. The study's aim was to independently test the SMART intervention that was originally developed by educational psychologists in Ireland at Maynooth University and determine whether it is effective for children with cerebral palsy.

I would like to ask you about your involvement with SMART to learn more about how other families might engage with the program.

Would it be possible to audio record our conversation so I can prepare an accurate transcript of the discussion?

The recording will be transcribed and stored securely, using your study ID rather than name, to maintain confidentiality.

Parent Questions:

1) What did you like best about the SMART program?

Follow-up: Why did you like X the best?

2) What did you like least about the SMART program?

Follow-up: "Why did you not like X?"

3) What do you think could have been different in the SMART program?

4) Did you experience any challenges in supporting your child in doing SMART?

5) Did you notice any changes in your child after participating in SMART? Follow-up: Have you noticed any changes with regard to schoolwork?

If so, what were the changes you noticed in your child after participating in SMART?

6) Did your child enjoy participating in SMART?

7)Have you noticed any changes in your relationship with your child since participating in SMART?

8) Would you participate in this program again?

9) Would you recommend SMART to other families?

10) Are you planning on continuing to use SMART at home? If so, why? If not, why not?

10) Do you have any further comments or suggestions about using SMART?

Questions for children:

1) What did you like best about the SMART program?

For peer review only

Follow-up: Why did you like X the best?

2) What did you like least about the SMART program?

Follow-up: Why did you not like X?

3) What do you think could have been different in the SMART program?

4) Was there anything that made it easy to use SMART at home? Manual? Parent involvement?

4) Can you tell me about anything that made it hard for you to use SMART at home? Other activities? Time?

5) Did you notice any changes in yourself after participating in SMART? If yes, ask what changes have you noticed after participating in SMART?

6) Did you enjoy participating in SMART?

7) Would you participate in this program again?

9) Would you recommend SMART to other children?

10) Do you think you'd keep using SMART? If so, why? If not, why not?

11) Is there anything else you'd like to tell me about SMART?

For peer review only

Queensland cerebral palsy & rehabilitation research centre

Queensland Government

THE UNIVERSITY OF QUEENSLAND AUSTRALIA

STANDARD INFORMED CONSENT - CHILD TO PARTICIPATE IN A RESEARCH PROJECT

Project Number

**HREC/14/QRCH/377**

Title of Project

A Randomised Controlled Trial of an Online Cognitive Rehabilitation Program for Children with Cerebral Palsy

Investigator(s)

Dr Koa Whittingham, Dr Jeanie Sheffield, Professor Roslyn Boyd, Ms Jane Wotherspoon

I (Parent/Guardian name) ______________________________

voluntarily consent for my child to take part in the above titled Research Project, explained to me by

Mr/Ms/Dr/Professor ______________________________

Child's Name ______________________________

Address ______________________________

Contact Phone Numbers ______________________________

I have received a Parent/Guardian Information Statement to keep and I believe I understand the purpose, extent and possible effects of my child's involvement

I have been asked if I would like to have a family member or friend with me while the project was explained

I have had an opportunity to ask questions and I am satisfied with the answers I have received

I understand that the researcher has agreed not to reveal results of any information involving my child, subject to legal requirements

If information about this project is published or presented in any public form, I understand that the researcher will not reveal my child's identity

I understand that if I refuse to consent to my child's participation, or if I withdraw my child from the project at any time without explanation, this will not affect my child's access to the best available treatment options and care from Children's Health Queensland.

If you do not wish to participate, **it is okay to say no**

I understand I will receive a copy of this consent form

- **I consent to participate in this research project.**

YES ☐ **NO** ☐

SIGNATURE ______________________________ Date ______________

I have explained the study to the parent/guardian who has signed above, and believe that they understand the purpose, extent and possible effects of their child's involvement in this study.

RESEARCHER'S SIGNATURE ______________________________ Date ______________

Note: All parties signing the Consent Form must date their own signature.

For peer review only - http://bmjopen.bmj.com/site/about/guidelines.xhtml

SPIRIT
STANDARD PROTOCOL ITEMS: RECOMMENDATIONS FOR INTERVENTIONAL TRIALS

SPIRIT 2013 Checklist: Recommended items to address in a clinical trial protocol and related documents*

Section/item	Item No	Description
**Administrative information**		
Title	1	Descriptive title identifying the study design, population, interventions, and, if applicable, trial acronym<br>**Page 1.**
Trial registration	2a	Trial identifier and registry name. If not yet registered, name of intended registry<br>**Page 3. This trial is registered with the Australian New Zealand Clinical Trials Registry, Trial ID: ACTRN12617001550392.**
	2b	All items from the World Health Organization Trial Registration Data Set<br>**NA**
Protocol version	3	Date and version identifier<br>**Page 20**
Funding	4	Sources and types of financial, material, and other support<br>**Page 20**
Roles and responsibilities	5a	Names, affiliations, and roles of protocol contributors<br>**Page 1, page 20**
	5b	Name and contact information for the trial sponsor<br>**NA, no sponsor**
	5c	Role of study sponsor and funders, if any, in study design; collection, management, analysis, and interpretation of data; writing of the report; and the decision to submit the report for publication, including whether they will have ultimate authority over any of these activities<br>**NA**
	5d	Composition, roles, and responsibilities of the coordinating centre, steering committee, endpoint adjudication committee, data management team, and other individuals or groups overseeing the trial, if applicable (see Item 21a for data monitoring committee)<br>**NA**
**Introduction**		

For peer review only

Background and rationale	6a	Description of research question and justification for undertaking the trial, including summary of relevant studies (published and unpublished) examining benefits and harms for each intervention
**Page 2, page 4-**		**Page 2, Page 4, page 9**
	6b	Explanation for choice of comparators<br>**Page 6, page 9**
Objectives	7	Specific objectives or hypotheses<br>**Page 9-10**
Trial design	8	Description of trial design including type of trial (eg, parallel group, crossover, factorial, single group), allocation ratio, and framework (eg, superiority, equivalence, noninferiority, exploratory)<br>**Page 10**

Methods: Participants, interventions, and outcomes

Study setting	9	Description of study settings (eg, community clinic, academic hospital) and list of countries where data will be collected. Reference to where list of study sites can be obtained<br>**Page 11**
Eligibility criteria	10	Inclusion and exclusion criteria for participants. If applicable, eligibility criteria for study centres and individuals who will perform the interventions (eg, surgeons, psychotherapists)<br>**Page 11**
Interventions	11a	Interventions for each group with sufficient detail to allow replication, including how and when they will be administered<br>**Page 18**
	11b	Criteria for discontinuing or modifying allocated interventions for a given trial participant (eg, drug dose change in response to harms, participant request, or improving/worsening disease)<br>**NA**
	11c	Strategies to improve adherence to intervention protocols, and any procedures for monitoring adherence (eg, drug tablet return, laboratory tests)<br>**Page 11**
	11d	Relevant concomitant care and interventions that are permitted or prohibited during the trial<br>**NA**

For peer review only - http://bmjopen.bmj.com/site/about/guidelines.xhtml

Outcomes	12	Primary, secondary, and other outcomes, including the specific measurement variable (eg, systolic blood pressure), analysis metric (eg, change from baseline, final value, time to event), method of aggregation (eg, median, proportion), and time point for each outcome. Explanation of the clinical relevance of chosen efficacy and harm outcomes is strongly recommended<br>**Page 13 to page 17**
Participant timeline	13	Time schedule of enrolment, interventions (including any run-ins and washouts), assessments, and visits for participants. A schematic diagram is highly recommended (see Figure)<br>**Flow Chart Figure 1 (CONSORT Flow chart)**
Sample size	14	Estimated number of participants needed to achieve study objectives and how it was determined, including clinical and statistical assumptions supporting any sample size calculations<br>**Page 18-19**
Recruitment	15	Strategies for achieving adequate participant enrolment to reach target sample size<br>**Page 11.**

Methods: Assignment of interventions (for controlled trials)

Allocation:

Sequence generation	16a	Method of generating the allocation sequence (eg, computer-generated random numbers), and list of any factors for stratification. To reduce predictability of a random sequence, details of any planned restriction (eg, blocking) should be provided in a separate document that is unavailable to those who enrol participants or assign interventions<br>**Page 11**
Allocation concealment mechanism	16b	Mechanism of implementing the allocation sequence (eg, central telephone; sequentially numbered, opaque, sealed envelopes), describing any steps to conceal the sequence until interventions are assigned<br>**Page 11**
Implementation	16c	Who will generate the allocation sequence, who will enrol participants, and who will assign participants to interventions<br>**Page 11-12**
Blinding (masking)	17a	Who will be blinded after assignment to interventions (eg, trial participants, care providers, outcome assessors, data analysts), and how<br>**Page 12 (no blinding)**

For peer review only - http://bmjopen.bmj.com/site/about/guidelines.xhtml

	17b	If blinded, circumstances under which unblinding is permissible, and procedure for revealing a participant's allocated intervention during the trial<br>**NA**

Methods: Data collection, management, and analysis

Data collection methods	18a	Plans for assessment and collection of outcome, baseline, and other trial data, including any related processes to promote data quality (eg, duplicate measurements, training of assessors) and a description of study instruments (eg, questionnaires, laboratory tests) along with their reliability and validity, if known. Reference to where data collection forms can be found, if not in the protocol<br>**Page 12, Page 13-17**
	18b	Plans to promote participant retention and complete follow-up, including list of any outcome data to be collected for participants who discontinue or deviate from intervention protocols<br>**Page 18**
Data management	19	Plans for data entry, coding, security, and storage, including any related processes to promote data quality (eg, double data entry; range checks for data values). Reference to where details of data management procedures can be found, if not in the protocol<br>**Page 12**
Statistical methods	20a	Statistical methods for analysing primary and secondary outcomes. Reference to where other details of the statistical analysis plan can be found, if not in the protocol<br>**Page 18**
	20b	Methods for any additional analyses (eg, subgroup and adjusted analyses)<br>**Page 18**
	20c	Definition of analysis population relating to protocol non-adherence (eg, as randomised analysis), and any statistical methods to handle missing data (eg, multiple imputation)<br>**Page 18**

Methods: Monitoring

Data monitoring	21a	Composition of data monitoring committee (DMC); summary of its role and reporting structure; statement of whether it is independent from the sponsor and competing interests; and reference to where further details about its charter can be found, if not in the protocol. Alternatively, an explanation of why a DMC is not needed<br>**Page 12**

For peer review only - http://bmjopen.bmj.com/site/about/guidelines.xhtml

	21b	Description of any interim analyses and stopping guidelines, including who will have access to these interim results and make the final decision to terminate the trial<br>**NA**
Harms	22	Plans for collecting, assessing, reporting, and managing solicited and spontaneously reported adverse events and other unintended effects of trial interventions or trial conduct<br>**Page 12**
Auditing	23	Frequency and procedures for auditing trial conduct, if any, and whether the process will be independent from investigators and the sponsor<br>**NA**
**Ethics and dissemination**		
Research ethics approval	24	Plans for seeking research ethics committee/institutional review board (REC/IRB) approval<br>**Page 19**
Protocol amendments	25	Plans for communicating important protocol modifications (eg, changes to eligibility criteria, outcomes, analyses) to relevant parties (eg, investigators, REC/IRBs, trial participants, trial registries, journals, regulators)<br>**Page 19**
Consent or assent	26a	Who will obtain informed consent or assent from potential trial participants or authorised surrogates, and how (see Item 32)<br>**Page 19**
	26b	Additional consent provisions for collection and use of participant data and biological specimens in ancillary studies, if applicable<br>**NA**
Confidentiality	27	How personal information about potential and enrolled participants will be collected, shared, and maintained in order to protect confidentiality before, during, and after the trial<br>**Page 12**
Declaration of interests	28	Financial and other competing interests for principal investigators for the overall trial and each study site<br>**Page 21**
Access to data	29	Statement of who will have access to the final trial dataset, and disclosure of contractual agreements that limit such access for investigators<br>**Page 20**

For peer review only - http://bmjopen.bmj.com/site/about/guidelines.xhtml

Ancillary and post-trial care	30	Provisions, if any, for ancillary and post-trial care, and for compensation to those who suffer harm from trial participation<br>**NA**
Dissemination policy	31a	Plans for investigators and sponsor to communicate trial results to participants, healthcare professionals, the public, and other relevant groups (eg, via publication, reporting in results databases, or other data sharing arrangements), including any publication restrictions<br>**Page 19**
	31b	Authorship eligibility guidelines and any intended use of professional writers<br>**Page 19**
	31c	Plans, if any, for granting public access to the full protocol, participant-level dataset, and statistical code<br>**NA**
**Appendices**		
Informed consent materials	32	Model consent form and other related documentation given to participants and authorised surrogates<br>**Appendix B**
Biological specimens	33	Plans for collection, laboratory evaluation, and storage of biological specimens for genetic or molecular analysis in the current trial and for future use in ancillary studies, if applicable<br>**NA**

*It is strongly recommended that this checklist be read in conjunction with the SPIRIT 2013 Explanation & Elaboration for important clarification on the items. Amendments to the protocol should be tracked and dated. The SPIRIT checklist is copyrighted by the SPIRIT Group under the Creative Commons "Attribution-NonCommercial-NoDerivs 3.0 Unported" license.

For peer review only

BMJ Open

A randomised controlled trial of a novel online cognitive rehabilitation program for children with cerebral palsy: A study protocol

Journal:	*BMJ Open*
Manuscript ID	bmjopen-2018-028505.R2
Article Type:	Protocol
Date Submitted by the Author:	11-May-2019
Complete List of Authors:	Wotherspoon, Jane; The University of Queensland, Queensland Cerebral Palsy and Rehabilitation Research Centre<br>Whittingham, Koa; The University of Queensland, Queensland Cerebral Palsy and Rehabilitation Research Centre<br>Boyd, Roslyn; The University of Queensland, Queensland Cerebral Palsy and Rehabilitation Research Centre<br>Sheffield, Jeanie; The University of Queensland, School of Psychology
**Primary Subject Heading**:	Paediatrics
Secondary Subject Heading:	Rehabilitation medicine
Keywords:	Developmental neurology < neurodisability < PAEDIATRICS, Developmental Psychology, Cognitive Impairment, Cognitive Training

SCHOLARONE™
Manuscripts

A randomised controlled trial of a novel online cognitive rehabilitation program for children with cerebral palsy: A study protocol

Researchers: Ms Jane Wotherspoon[1]

Dr Koa Whittingham[1] (Chief Investigator)

Professor Roslyn N. Boyd, PhD[1,2]

Dr Jeanie Sheffield[2]

Affiliations: [1]Queensland Cerebral Palsy and Rehabilitation Research Centre (QCPRRC), The University of Queensland

[2]Faculty of Medicine, The University of Queensland

[3]School of Psychology, The University of Queensland

Correspondence to Jane Wotherspoon, Queensland Cerebral Palsy and Rehabilitation Research Centre (QCPRRC), 62 Graham Street, South Brisbane, Queensland, 4101. E-mail: j.wotherspoon@uq.edu.au

For peer review only - http://bmjopen.bmj.com/site/about/guidelines.xhtml

Abstract

Introduction

Cerebral palsy (CP) is the most common cause of physical disability in children, with an estimated 600-700 infants born with CP in Australia each year. Cerebral palsy is typically associated with motor impairments, but nearly half of all children with CP also experience cognitive impairment, potentially impacting educational and vocational achievement. This paper reports the protocol for a randomised controlled trial of a computerised cognitive training intervention based on behavioural principles, Strengthening Mental Abilities through Relational Training (SMART). The study aims to investigate SMART's effect on fluid reasoning, executive function and academic achievement in children with CP.

Methods and Analysis

Sixty children with mild to moderate CP (Gross Motor Function Classification Scale – GMFCS I - IV) aged between 8 and 12 will be recruited. Participants will be randomly allocated to two groups: SMART cognitive training and waitlist control. Families will access the program at home over a four-month period. Assessments will be administered at baseline, 20 weeks and at 40-week follow-up for retention. The primary outcome will be fluid intelligence, while academic achievement, executive function and social and emotional wellbeing will be secondary outcomes.

Ethics and Dissemination

This study has approval from the Children's Health Queensland Hospital and Health Service Research Ethics Committee (HREC/14/QRCH/377) and The University of Queensland (2017001806). If the computerised cognitive training program is found to be effective, dissemination of these findings would assist children with CP by providing an accessible, cost-effective intervention that can be completed at home at the individual's own pace.

For peer review only - http://bmjopen.bmj.com/site/about/guidelines.xhtml

Registration Details

This trial is registered with the Australian New Zealand Clinical Trials Registry, Trial ID: ACTRN12617001550392. The study was registered prospectively on 10 November 2017 to present. Recruitment is now under way and we aim to complete recruitment by June 2019, with data collection finalised by March 2020.

Strengths and limitations of this study

- This is the first study to trial the effectiveness of a computerised cognitive intervention in maximising fluid intelligence in children with cerebral palsy.
- It is the first randomised controlled trial of a computerised cognitive intervention based on relational frame theory for children with a developmental disability.
- Interventions for cognitive impairment in this population are not readily available, and if effective, this intervention would provide a cost-effective, easily accessible intervention.
- All participants will receive access to the computerised cognitive training intervention prior to the end of the study.
- No active control group is included in this study; therefore we cannot determine impact of the intervention independent of potential placebo or expectancy effects arising from focused use of a computer program.

Introduction

Cerebral palsy (CP), with a prevalence of 1.4 per 1000 live births,[1] is the most common cause of physical disability in children and an estimated 600-700 infants are born with CP in Australia each year.[2] While CP is typically associated with motor impairments, research is now focusing on accompanying cognitive and executive functioning (EF) deficits and how

For peer review only - http://bmjopen.bmj.com/site/about/guidelines.xhtml

these impact daily living.[3] It is estimated that approximately 45% of children with CP will have an intellectual impairment impacting significantly on educational achievement.[4-7] A number of studies have found cognitive impairment in CP is associated with long-term difficulties in completing formal education, obtaining competitive employment and living independently.[8-11]

While there is growing awareness of cognitive and EF limitations, interventions for CP are typically associated with improving physical activity, limb function, and participation in daily living activities.[12] Few interventions target cognitive function and academic abilities.[12, 13] A review looking at new technologies in the treatment of CP and developmental coordination disorder found no studies investigating specific cognitive interventions in these groups. [14] A small number of prior studies have examined whether aspects of cognitive function can be improved, through either web-based multimodal therapy ("Move it to Improve it" [Mitii])[15] or a mindfulness-based yoga programme (MiYoga)[16], A randomised controlled trial of Mitii measured visual perceptual skills of participants and found a significant effect of the web-based therapy program on visual-perceptual skill, although concluded that the effect was not of clinical significance. [15] Mak et al. found a mindfulness-based movement intervention demonstrated significant better sustained attention post-intervention than a waitlist control group, but no differences were found for other measures associated with cognition, including working memory and executive function. [16]

For children with CP who live in remote or isolated communities, access to clinic-based interventions is further limited. This study aims to trial a novel online cognitive rehabilitation program for children with CP targeted to improve intellectual functioning, EF, and educational achievement. The program - Strengthening Mental Abilities Through Relational Training (SMART)[17] - is founded upon relational frame theory, which proposes

For peer review only - http://bmjopen.bmj.com/site/about/guidelines.xhtml

that the development of language and complex reasoning in humans rests upon our ability derive relations between stimuli arbitrarily and without direct experience. An online program designed to train relational framing ability and potentially improve complex reasoning would be a cost-effective, accessible intervention for children with CP.

Cerebral palsy and cognitive impairment

Cerebral palsy refers to a group of motor disorders, originating in a non-progressive injury or disturbance to the brain.[18-20] These disturbances occur early in development, impacting the foetal or infant brain.[19] Cerebral palsy is associated with various types of brain lesions, [21] arising from many different causes, congenital and acquired, including intracranial haemorrhage, asphyxia, prematurity, low birth weight or infection.[22]

While motor impairment is a defining feature of CP, more recent definitions acknowledge the frequent comorbidities, such as vision problems, epilepsy, and cognitive and communication difficulties.[22, 23] Fewer studies have focused on cognitive impairment in cerebral palsy than on motor impairment, but the research that has been undertaken suggests a significant proportion of individuals with CP have impaired cognitive function.[7] A systematic review of rates of impairment in CP found that 49% of children with CP had an intellectual disability, defined as an IQ below 70, while 28% had a severe intellectual disability, with an IQ below 50.[21] Similarly, a population-based study of more than 1,100 individuals with CP in Australia found 45% had been recorded as having an intellectual disability, although level of impairment could not be determined for many in this sample.[7] It is recognised that although CP is a non-progressive disease, the impact of these additional impairments can exert significant influence over a child's development, impacting academic and vocational outcomes, psychological well-being and quality of life,[21] as well as general health.[7]

For peer review only - http://bmjopen.bmj.com/site/about/guidelines.xhtml

Fluid Intelligence

Amongst most intelligence researchers, there is broad agreement that intelligence is associated with certain abilities, such as problem solving, understanding abstract ideas, or capacity for learning.[24-26] One prominent contemporary model, and the most influential at present in the field of intelligence testing, is the Cattell-Horn-Carroll (CHC) theory of intelligence.[27, 28] CHC theory offers a system for classifying cognitive ability that has allowed greater consensus in the literature around what is being measured and referred to by terms such as crystallised and fluid intelligence.[28] Fluid intelligence is a measure of the capacity to solve novel problems and reason abstractly, while crystallised intelligence is a measure of comprehension and acquired knowledge.[29]

While in the past, intelligence was considered a stable attribute, more recently, it has been conceptualised as a quality open to change and development.[30] As a result, attention has turned to potential factors that could play a role in determining intelligence, with the aim of developing interventions.[31] Furthermore, by combining technological innovation with proposed models of cognitive plasticity, the possibility of accessible interventions, delivered via computers, iPads or similar devices, has emerged.[14] For example, Løhaugen et al.[32] have proposed a randomised controlled trial assessing the efficacy of computer-based working memory training in children with cerebral palsy.

Various online cognitive training programs have been developed and many of these have targeted working memory (WM), an executive function that involves the ability to temporarily hold and manipulate information. Some researchers have hypothesised that gains in WM could transfer into gains in fluid intelligence.[33, 34] Research into this area is ongoing, and results are mixed.[33] While Jaeggi et al.[34] generated great interest in cognitive

For peer review only - http://bmjopen.bmj.com/site/about/guidelines.xhtml

training when they reported gains in fluid intelligence after WM training,[35] numerous studies have failed to replicate such transfer effects,[36] and research continues in this field.

SMART program

The novel online cognitive training program to be trialled, Strengthening Mental Abilities through Relational Training (SMART), shows promise in pilot studies in helping improve children's cognitive skills (e.g., ability to learn, think, and reason).[17] SMART is a web-based cognitive training program, currently available in English or Dutch, that directly trains the relational abilities thought to be foundational to complex cognition. SMART is grounded in contextual behavioural science, specifically Relational Frame Theory,[17] which proposes that all human language and complex cognition is underpinned by our ability to relate stimuli arbitrarily**.** That is, humans can relate stimuli in a manner that does not correspond to the physical properties of the stimuli. Such relations between stimuli are called relational frames, and a number of such frames exist, such as coordination (e.g., same as), comparative (e.g., more/less), and temporal frames (e.g., before and after).[37] An example of a relational frame of more/less would be with Australian one- and two-dollar coins, where individuals learn that the two-dollar coin is worth more, even though it is physically smaller than the one-dollar coin.

Many relational frames, including spatial (e.g. under), measurement (e.g. bigger) and ordinal (e.g. first) are foundational to mathematics.[38] Repeated exposure to spatial, measurement, ordinal and other mathematics-relevant relational frames is considered a critical part of early education, helping students develop their understanding of mathematical concepts.[38]

In addition to learning relational frames, people can also learn behavioural responses through derived relations, not merely through direct experience.[17] Through derived relations, our relational framing abilities greatly enhance our capacity to learn and to interact effectively with others and our environment. As an example, a person might learn that the star Vega is

For peer review only - http://bmjopen.bmj.com/site/about/guidelines.xhtml

closer to Earth than Canopus, but further than Sirius. They could then derive that Canopus is the furthest star from Earth of the three, even though it has not been directly taught, and Canopus appears brighter than Vega. As relational framing is foundational to complex cognition, the direct training of relational framing itself has the potential to have wide-reaching effects on cognition.

The SMART program itself consists of 55 modules that can be worked through at the participant's own pace. Progress to each module requires successful completion of the preceding one. A maximum of five modules can be completed per day. Each module presents a proposition in the form of a relations between nonsense words, and then asks a yes/no question based on the proposition. For example, "SAJ is the same as MIS. Is MIS the same as SAJ?". Derived relations are also trained through the addition of more than two nonsense words. For example, "SAJ is the same as MIS. QUW is the same as SAJ." Is QUW the same as MIS?" Each module provides multiple examples of the relationship being trained, and if 16 questions are answered correctly, each within a 30-second time frame, the next module is unlocked. Additional relations trained include *opposite*, *more than* and *less than*.

A feasibility pilot study of SMART was conducted over a 9-month period with eight children aged 11-12 years old who had been experiencing educational difficulties at school.[17] Seven of the eight children showed significant increases in their intellectual functioning (as measured by the Wechsler Intelligence Scale for Children – Fourth Edition), an improvement of more than one standard deviation. A 2016 study found similar significant increases in IQ, with a sample size of 15 11- to 12-year-old children. [31] While promising, practice effects need to be accounted for when repeated administration of standardised measures of intelligence occurs, as they may influence performance, with average gains of 6-7 points over a one-month period found for the WISC-V measure of full-scale IQ [39]. Furthermore, assessment of fluid reasoning ability may be more affected by practice effects than verbal or working memory

For peer review only - http://bmjopen.bmj.com/site/about/guidelines.xhtml

tasks [39], as fluid reasoning tasks are associated with ability to solve novel problems. A randomised controlled design rather than pre- and post-intervention studies could control for practice effects, but the two studies that have utilised this design have been limited by small sample sizes and high attrition rates. [40, 41] To date, no studies have investigated the efficacy of SMART in the cerebral palsy population.

The underlying cognitive skills trained in the program are required for vocabulary acquisition, mathematical reasoning, and other academic and learning skills. The program can be accessed in the client's home at their convenience via iPad, Mac or PC with Internet access and can be completed at the child's own pace. As such, it is a potentially cost-effective solution that can be delivered to children with CP who are unable to access ongoing rehabilitation services.

METHODS

Specific Aims

The aim of this randomised controlled trial is to test the efficacy of a novel web-based cognitive rehabilitation program for children aged between 8 and 12 years old with mild to moderate congenital CP.

Hypotheses

The primary hypothesis to be tested is that in a randomised controlled trial for children aged 8-12 with CP:

1. Participants in the intervention group will demonstrate improved performance on a standardised test of intellectual ability immediately post-intervention when compared to a waitlist control group receiving care as usual. (Wechsler Intelligence Scale for Children - Fifth Edition; WISC-V)[42, 43]

The secondary hypotheses to be tested are that the SMART intervention group will demonstrate improvements in the following outcomes:

For peer review only - http://bmjopen.bmj.com/site/about/guidelines.xhtml

1. Academic achievement (Wechsler Individual Achievement Test Third Edition; WIAT-III)[44]

2. Executive function (Behavior Rating Inventory of Executive Function; BRIEF)[45]

3. Social and emotional functioning (Strengths and Difficulties Questionnaire; SDQ; Behavior Assessment System for Children - Third Edition; BASC-3; Social Communication Questionnaire; SCQ;)[46-48].

4. Attention (Conners - Third edition; Conners-3)[49].

5. Quality of life (Cerebral Palsy Quality of Life - Child; CP-QOL)[50].

Study Design

The study is a randomised controlled trial design with waitlist control group, to determine the effectiveness of the SMART program for children with CP aged 8-12 years (see CONSORT flowchart in Figure 1.) After baseline assessment, participants will be randomly allocated to either the intervention or waitlist control group.

Outcomes will be assessed for all participants at three timepoints, baseline, at 20 weeks post-baseline, and at 40 weeks post-baseline. The intervention group will commence the SMART program immediately and complete it over the following 20 weeks, before undergoing post-intervention assessment at 20 weeks post-baseline and follow-up assessment at 40 weeks post-baseline. Participants will be provided with log-in details enabling them to access to the online program at no cost via the program website (http://raiseyouriq.com/) for up to five months. They will be able to access it at home via either iPad or computer. The waitlist control group will continue care as usual for 20 weeks before returning for a second assessment visit, at which point they will commence the SMART program. Final assessment for the waitlist control group will be post-intervention at 40 weeks.

Participants: We aim to recruit 60 children with mild to moderate CP (Gross Motor Function Classification Scale – GMFCS I - IV) aged between 8 and 12 years old. All children are

For peer review only - http://bmjopen.bmj.com/site/about/guidelines.xhtml

required to have sufficient cooperation and cognitive understanding to perform the tasks and access the novel online cognitive training program, have access to the internet at home, and be able to attend three assessment sessions in Brisbane. Sufficient cooperation and cognitive understanding will be confirmed at baseline assessment, as participants who are able to undertake the iPad-based assessments will be deemed to meet criteria. Children will be excluded if they have unstable epilepsy (i.e., not controlled by medication); an unstable brain injury (e.g., degenerative or metabolic condition); and/or active medical condition (e.g., chemotherapy, radiotherapy, or neurosurgical).

Recruitment: Participants will be recruited from a consent-based research database at the Queensland Cerebral Palsy and Rehabilitation Research Centre, and through the Queensland Paediatric Rehabilitation Service at the Queensland Children's Hospital. Participants will be enrolled in the study by the first author.

Patient and public involvement

Participants, families and the public were not involved in the design or recruitment of this study. Participants are informed of the study burden from the time of initial contact, and are advised of their ability to withdraw from the study at any time. All participants will receive feedback on the results of the assessments administered once they have completed their involvement in the study.

Randomisation

Baseline assessments and demographic questionnaires will be completed prior to randomisation. Once complete, participants will be randomised to either waitlist control or intervention group. Randomisation will be via stratified random blocks, using a computer-generated block randomisation sequence, stored by staff members unconnected with the study. Allocation to either waitlist control or intervention will be recorded on pieces of paper, and these will be folded, then placed inside opaque envelopes by a staff member not involved

For peer review only

in the study. Envelopes will be sealed, and only opened upon completion of baseline assessment. Participants will be stratified according to IQ (<70 or ≥70), as measured on baseline assessment.

Blinding

Given the nature of the intervention, participants will not be blinded as to which group they are assigned to. As assessment will be undertaken by the first author as part of a PhD project, assessors will not be blinded in this project.

Adverse events

No health or safety risks associated with participation in this study have been identified, and the risk of adverse events is considered low. Any events associated with either intervention or wait-list control groups over the course of the study will be recorded. All participants allocated to the wait-list control group receive access to the intervention after the second assessment. This ensures no adverse impacts through omission of intervention in the event that the intervention is found to be efficacious. If any adverse events are identified, they will be reviewed by the investigators. As the ethical review process and trial conduct is overseen by two ethics committees, no additional data monitoring is considered necessary.

Data Management

Participants will be allocated randomly generated identification codes, and these will be used to de-identify hardcopy and electronic files. Paper copies relating to assessment will be de-identified and physically stored in a locked filing cabinet at the Queensland Cerebral Palsy and Rehabilitation Research Centre. Electronic data will be stored on REDCap, a secure web platform for creating and managing online databases. The installation of REDCap used for this project is hosted bv the University of Queensland and managed by the Queensland Clinical Trials and Biostatistics Centre.

For peer review only - http://bmjopen.bmj.com/site/about/guidelines.xhtml

Measures:

Demographic information will be obtained via a parent survey, gathering information on the participant's background, including gestational age, comorbid diagnoses, and GMFCS classification. Further demographic information includes school year, type of school, and whether any additional teaching support is accessed, along with parent education and household income.

All children will undergo a comprehensive cognitive, psychoeducational, and psychosocial assessment by a psychologist at baseline (i.e., before treatment), and re-assessment at 20 weeks (after intervention for immediate group), and 40 weeks (retention for immediate group, and after intervention for control group). It is noted that many of these assessments have not been validated for children with cerebral palsy, and have been chosen as no valid alternatives are available. However a review of assessments for children with cerebral palsy found that motor involvement, communication and visual impairment were key factors in determining suitability of assessments. [51] We have specifically chosen an iPad-based assessment delivery format for our primary outcome (full-scale IQ), that is similar in motor and language demand to the intervention program itself. If children are able to meet the inclusion criteria for the study, it is anticipated that they will also be able to complete the assessments.

Children will complete the following assessments:

- *The Wechsler Intelligence Scale for Children Fifth Edition (WISC-V)* is a standardised measure of overall cognitive/intellectual functioning. The WISC-V produces a full-scale intelligence IQ score (FSIQ), along with five primary index scores – Verbal Comprehension Index (VCI), Visual-Spatial Index (VSI), Fluid Reasoning Index (FRI), Working Memory Index (WMI), and Processing Speed Index (PSI), each derived from two subtests. The WISC-V has been found to have good internal consistency (α=.96 for FSIQ and α= .86-.94 for primary index scores).[43] Interrater

For peer review only

reliability has also been found to be acceptable (*r*= .98-.99 for interscorer agreement on a subset of subtests).[42]

- *The Wechsler Individual Achievement Test Third Edition (WIAT-III)* is a standardised measure of educational achievement. It provides measures of achievement across domains of oral language, reading, written language and mathematics. The internal consistency coefficients are good (α=.83-.95), with the exception of a single subtests (Alphabet Writing Fluency, α=.69), which is not used with the age range participating in this study.[44]
- *The Strengths and Difficulties Questionnaire (SDQ) - Child self-report*. This questionnaire measures the child's behaviour and adjustment. The SDQ is a 25-item measure with frequency of behaviour rated on a 3-point Likert scale.[46] The SDQ produces five subscales: emotional symptoms, conduct problems, inattention/hyperactivity, peer problems and prosocial behaviour (range 0-10), and a total difficulties score (range 0-40). Research suggest that for younger children, internal consistency is acceptable for the total difficulties, emotional symptoms, prosocial behaviour, and inattention/hyperactivity subscales, but not for peer and conduct problems.[52] However, Muris et al.[52] note that the scale can provide useful information about psychopathology in children from eight years old.

Parents will also be administered questionnaires during the three assessments points. These include:

- *The Behaviour Rating Inventory of Executive Function (BRIEF)* assesses the child's executive functioning in everyday life. The *BRIEF* is an 85-item parent-rated questionnaire assessing behavioural manifestations of executive functions in everyday life.[45] The *BRIEF* produces a Behavioural Regulation Index (BRI; Initiate, Working

For peer review only - http://bmjopen.bmj.com/site/about/guidelines.xhtml

Memory, Plan/Organise, Organisation of Materials, and Monitor subscales) a Metacognition Index (MCI; Inhibit, Emotional Control, and Shift subscales). The *BRIEF* has been shown to be a valid measure of executive functioning and has good internal consistency (α=.80-.98) and high test-retest reliability on the BRI (r=.92), MCI (r=.88), and the GEC (r=.86).

- *Conners-3 Parent Report,* which assesses childhood behaviours and behavioural disorders including Attention Deficit Hyperactive Disorder in children and adolescents aged 6-18.[49] The Conners-3 consists of 110 statements and takes approximately 20 minutes to complete. The Conners-3 measures seven key areas – inattention, learning problems, aggression, family relations, hyperactivity/impulsivity, executive functioning, and peer relations. Both internal consistency coefficients (α=.83-.94) and test-retest reliability (r=.52-.94) are good.
- The *Behavior Assessment System for Children – 3rd Edition (BASC-3)* measures children's adaptive and problem behaviours and emotional difficulties at home and in community settings [48]. It is completed by a parent or guardian and consists of 175 statements, taking approximately 20 minutes. The *BASC-3* produces five composite scales, Internalizing Problems, Externalizing Problems, School Problems, Adaptive Skills and the Behavioural Symptoms Index. Internal consistency coefficients for the Parent Rating Scale (α=.79-.97 for 6- to 7-year-olds; α=.79-.96 for 8- to 11-year-olds) and test-retest reliability (r=.80-.92 for Child scales) are good.
- *Social Communication Questionnaire – Current, (SCQ - Current)* screens for communication skills and social functioning difficulties associated with autism spectrum disorders. The *SCQ – Current* evaluates communication skills and social functioning in children, based on the previous three months. While research suggests the SCQ – Current has lower internal consistency than the Lifetime version,[53]

For peer review only - http://bmjopen.bmj.com/site/about/guidelines.xhtml

concerns around its use particularly focus on children below five years of age, which is outside the age range of participants in this study, and on using the Current form as an alternative to the Lifetime form for screening for Autism Spectrum Disorder. For the purpose of measuring change over time, however, the Current form is recommended.[47] It contains 40 items and is completed by a parent or primary caregiver in under 10 minutes. Scores on the SCQ provide information on the following domains: Social Interaction, Communication and Stereotyped, Repetitive or Restricted Behaviour.

- *Strengths and Difficulties Questionnaire (SDQ) – Parent Report* measures pro-social, internalising and externalising behaviours in children. It is a 25-item measure with frequency of behaviour rated on a 3-point Likert scale. The *SDQ* is a 25-item parent-report measure of child behaviour and adjustment, with frequency of behaviour rated on a 3-point Likert scale.[46] The SDQ produces five subscales: emotional symptoms, conduct problems, inattention/hyperactivity, peer problems and prosocial behaviour (range 0-10). It produces a total difficulties score (range 0-40) found to have adequate internal reliability (α=.73) and test-retest reliability (r=.62) as well as discriminant and concurrent validity.[46]
- *Cerebral Palsy Quality of Life for Children- Parent Report (CP-QOL-Child)* measures child quality of life. *CP QOL-Child* is a 66-item parent-report measure of child quality of life specifically developed for use in children with CP.[50] It measures quality of life across domains including physical wellbeing, social wellbeing, emotional wellbeing, school, service access and social acceptance. It has good concurrent validity, internal consistency (α=.76-.89) and test-retest reliability (r=.80-.90).[50]

For peer review only - http://bmjopen.bmj.com/site/about/guidelines.xhtml

The SMART program provides an internal measure of Relational Ability, and all children will complete this measure at baseline, 20 weeks and 40 weeks. In addition, information will be recorded on time taken for each participant to complete the program. Assessments at baseline, 20 and 40 weeks will take approximately two to three hours per session. The waitlist control design ensures all children in the study will receive the intervention within six months of being randomised to either commence the program immediately or after 20 weeks. Table 1 summarises the measures for each assessment point.

**Measures** (T1, T2, T3)
**Child:**<br>Intellectual Functioning (WISC-V)<br>Academic Achievement (WIAT-III)<br>Social-Emotional Functioning (SDQ – Child self-report)<br>Relational Ability (RAI)<br><br>**Parent:**<br>Executive Function (BRIEF)<br>Attention and Behaviour (Conners-3)<br>Social-Emotional Functioning (BASC-3, SDQ – Parent report)<br>Social Communication (SCQ –Current)<br>Quality of Life (CP-QOL Child – Parent report)

Table 1. Summary of Assessment Measures.

Qualitative Interview

At the conclusion of the study, semi-structured interviews will be conducted with children and caregivers by the first author, a registered psychologist. The aim of the interview is to explore participants' engagement with the online cognitive rehabilitation program and gain qualitative insights into families' experience with the program. Questions will cover what families like and disliked about the program, how easy they found it was to access at home, and to remain engaged. If the program is found to be effective, such qualitative insights will

For peer review only - http://bmjopen.bmj.com/site/about/guidelines.xhtml

be valuable in the translation phase. The script for the interview can be found in Supplementary Appendix A. Interviews will be recorded, transcribed and analysed.

Intervention: With the SMART online platform, participants answer problems directly training relational framing and receive immediate feedback on their answers. Study participants are provided with an alias login name (to maintain confidentiality) and a password. Children are encouraged to complete the SMART intervention for 30 minutes per session, for a total of 1.5 hours per week. SMART is incremented and can be completed at the child's own pace. The full dosage is reached when the child completes the entire intervention, which is expected to occur within 20 weeks. Throughout the RCT, a psychologist will regularly contact the family and monitor that child's progress, supporting both parent and child in meeting their goals for SMART, and maintaining engagement.

A resource guide prepared for this study will be provided to all families. This guide provides technical information on accessing the program, a description of how to work through each stage of the program, and information on how parents can support their child to work on the program, including a visual chart to keep track of progress.

Statistical Analysis: Study hypotheses will be analysed by means of appropriate statistical tests, with statistical significance for all tests set at $p<0.05$ with adjustment for multiple comparisons, and all analyses will be intention to treat. We propose to carry out a Benjamini-Hochberg procedure to control for false discoveries.[54]

Mixed analysis of covariance analyses will be conducted with time (baseline and 20 weeks) as the within subjects variable, and group (intervention or waitlist) as the between subjects variable, and baseline data as the covariate. Secondary analysis will profile cognitive change over time for participants based on their test scores. This will include *t* tests and linear

For peer review only - http://bmjopen.bmj.com/site/about/guidelines.xhtml

regression to explore within-subject differences from post-intervention to follow-up, and over three timepoints (baseline, post-intervention and follow-up) for participants in the intervention group.

Power Analysis and Sample Size:

Power analysis was conducted using the software package G*Power [55], for an ANCOVA repeated measures, between factors test. With a sample size of 60 subjects, and assuming an error rate of 5% and p=0.70 for within-subject correlations, this sample size results in 81.37% power to detect a large (Cohen's f=0.40) mean difference of 12 IQ points between groups, after allowing for an attrition rate of 10%.

Ethics and Dissemination

This study has received full ethical approval from the Children's Health Queensland Hospital and Health Service Research Ethics Committee (HREC/14/QRCH/377) and The University of Queensland (2017001806). Protocol modifications and amendments will be submitted to the ethics committees for approval. Amendments to the protocol will be included in publications reporting on outcomes of this study. All families will be provided with a written informed consent form by the first author at the initial visit (Supplementary Appendix B) that they will be required to sign before commencing participation. This trial has been registered with the Australian New Zealand Clinical Trials Registry, Trial ID: ACTRN12617001550392. Study results will be disseminated through publication in scientific journals and participation in conferences. The authors of this protocol will be authors of any publications describing study outcomes, and professional writers will not be used. Families who participate in the study will receive information on the study results, as well as a feedback report on the outcomes of assessments their child has completed. If the computerised cognitive training program is found to be effective, dissemination of these

For peer review only - http://bmjopen.bmj.com/site/about/guidelines.xhtml

For peer review only

findings would assist children with CP by providing an easily accessible, cost-effective intervention that can be completed at home at the individual's own pace.

Trial Progress

This protocol is Version 3, 10 May 2019. The study is currently actively recruiting participants, after initial recruitment commenced in June 2018.

Discussion

This protocol paper has reported the background and study design for a randomised controlled trial investigating the effectiveness of a computerised cognitive training program for children with CP. This program has not previously been studied in this population. The research study will assess children's cognitive skills, executive ability and social and emotional functioning, with fluid intelligence the primary outcome of interest. Qualitative information will be gathered on families' experience engaging with the program. Results of the study will be disseminated through peer-reviewed journals and at relevant scientific conferences.

Nearly half of all children with CP are estimated to also have an intellectual impairment, impacting academic achievement and ability to achieve educational and vocational goals in the long-term. If this computerised cognitive training program is found to be effective, a flexible, easily accessible intervention will be available for this population, where at present there at few options available for addressing difficulties with cognitive skills in CP.

Contributors

The study was designed and established by all the authors. JW is responsible for the ethics application and reporting. JW is responsible for recruitment and data collection. JW will take a lead role in preparing publications on the clinical outcomes of the study. KW, RB and JS

For peer review only - http://bmjopen.bmj.com/site/about/guidelines.xhtml

For peer review only

will contribute to the preparation of publications and are providing supervision throughout the study. JW will take on a lead role for statistical analysis. JW drafted the final version of this manuscript, while all authors critically reviewed and approved the final version. JW will use data from this study to contribute to her PhD thesis.

Funding

JW is a PhD scholar funded by an Australian Government Research Training Program Stipend and Queensland Cerebral Palsy and Research Rehabilitation Centre Top-up Scholarship.

Competing Interests

The authors state they have no competing interests in this project.

For peer review only

References

1. Group ACPR. Report of the Australian Cerebral Palsy Register Birth years 1995-2012, November 2018 2018.

2. Economics A. The economic impact of cerebral palsy in Australia in 2007. Canberra: Access Economics; 2008.

3. Bottcher L, Flachs EM, Uldall P. Attentional and Executive Impairments in Children with Spastic Cerebral Palsy. Developmental Medicine & Child Neurology. 2010;52(2):e42-e7.

4. Himmelmann K, Beckung E, Hagberg G, Uvebrant P. Gross and fine motor function and accompanying impairments in cerebral palsy. Developmental Medicine & Child Neurology. 2006;48(6):417-23.

5. Rankin J, Cans C, Garne E, Colver A, Dolk H, Uldall P, et al. Congenital anomalies in children with cerebral palsy: a population-based record linkage study. Developmental Medicine & Child Neurology. 2010;52(4):345-51.

6. Andersen GL, Irgens LM, Haagaas I, Skranes JS, Meberg AE, Vik T. Cerebral palsy in Norway: Prevalence, subtypes and severity. European Journal of Paediatric Neurology. 2008;12(1):4-13.

7. Reid SM, Meehan EM, Arnup SJ, Reddihough DS. Intellectual disability in cerebral palsy: a population-based retrospective study. Developmental Medicine & Child Neurology. 2018;60(7):687-94.

8. Benner JL, Hilberink SR, Veenis T, van der Slot WMA, Roebroeck ME. Course of employment in adults with cerebral palsy over a 14-year period. Developmental Medicine & Child Neurology. 2017;59(7):762-8.

For peer review only

9. Mesterman R, Leitner Y, Yifat R, Gilutz G, Levi-Hakeini O, Bitchonsky O, et al. Cerebral Palsy—Long-Term Medical, Functional, Educational, and Psychosocial Outcomes. Journal of Child Neurology. 2009;25(1):36-42.

10. Michelsen SI, Uldall P, Kejs AMT, Madsen M. Education and employment prospects in cerebral palsy. Developmental Medicine & Child Neurology. 2005;47(8):511-7.

11. Murphy KP, Molnar GE, Lankasky K. Employment and social issues in adults with cerebral palsy. Archives of Physical Medicine and Rehabilitation. 2000;81(6):807-11.

12. Novak I, McIntyre S, Morgan C, Campbell L, Dark L, Morton N, et al. A systematic review of interventions for children with cerebral palsy: state of the evidence. Developmental Medicine & Child Neurology. 2013;55(10):885-910.

13. Løhaugen GCC, Beneventi H, Andersen GL, Sundberg C, Østgård HF, Bakkan E, et al. Do children with cerebral palsy benefit from computerized working memory training? Study protocol for a randomized controlled trial. Trials. 2014;15(1):269.

14. Wilson P, Green D, Caeyenberghs K, Steenbergen B, Duckworth J. Integrating New Technologies into the Treatment of CP and DCD. Current Developmental Disorders Reports. 2016;3(2):138-51.

15. James S, Ziviani J, Ware RS, Boyd RN. Randomized controlled trial of web-based multimodal therapy for unilateral cerebral palsy to improve occupational performance. Developmental Medicine & Child Neurology. 2015;57(6):530-8.

16. Mak C, Whittingham K, Cunnington R, Boyd RN. Effect of mindfulness yoga programme MiYoga on attention, behaviour, and physical outcomes in cerebral palsy: a randomized controlled trial. Developmental Medicine & Child Neurology. 2018;60(9):922-32.

17. Cassidy S, Roche B, Hayes SC. A relational frame training intervention to raise intelligence quotients: A pilot study. The Psychological Record. 2011;61(2):173-98.

For peer review only

For peer review only - http://bmjopen.bmj.com/site/about/guidelines.xhtml

18. Badawi N, Watson L, Petterson B, Blair E, Slee J, Haan E, et al. What constitutes cerebral palsy? Developmental Medicine & Child Neurology. 1998;40(8):520-7.

19. Rosenbaum P. The Definition and Classification of Cerebral Palsy: Are We Any Further Ahead in 2006? NeoReviews. 2006;7(11):e569-e74.

20. Smithers-Sheedy H, Badawi N, Blair E, Cans C, Himmelmann K, Krägeloh-Mann I, et al. What constitutes cerebral palsy in the twenty-first century? Developmental Medicine & Child Neurology. 2014;56(4):323-8.

21. Novak I, Hines M, Goldsmith S, Barclay R. Clinical prognostic messages from a systematic review on cerebral palsy. Pediatrics. 2012;130(5):e1285.

22. Straub K, Obrzut J. Effects of Cerebral Palsy on Neuropsychological Function. Journal of Developmental and Physical Disabilities. 2009;21(2):153-67.

23. Rosenbaum P, Paneth N, Leviton A, Goldstein M, Bax M, Damiano D, et al. A report: the definition and classification of cerebral palsy April 2006. Developmental Medicine & Child Neurology. 2007;49(109):8-14.

24. Carr A, O'Reilly G, Walsh PN, McEvoy J. The Handbook of Intellectual Disability and Clinical Psychology Practice. Hoboken: Routledge; 2014.

25. Demetrious A. Tracing psychology's invisible giant and its visible guards. In: Sternberg RJ, & Grigorenko, E.L., editor. The General Factor of Intelligence: How general is it? New York: Psychology Press; 2012. p. 3-18.

26. Gottfredson LS. Mainstream science on intelligence: An editorial with 52 signatories, history, and bibliography. 1997. p. 13-23.

27. Ortiz SO. CHC Theory of Intelligence. In: Goldstein S, Princiotta D, Naglieri JA, editors. Handbook of Intelligence: Evolutionary Theory, Historical Perspective, and Current Concepts. New York, NY: Springer New York; 2015. p. 209-27.

For peer review only - http://bmjopen.bmj.com/site/about/guidelines.xhtml

28. Schneider WJ, McGrew KS. The Cattell-Horn-Carroll Theory of Cognitive Abilities. In: Flanagan DP, McDonough EM, editors. Contemporary Intellectual Assessment:Theories, Tests, and Issues. Fourth ed. New York: The Guildford Press; 2018. p. 73-163.

29. Keith TZ, Reynolds MR. Cattell-Horn-Carroll Abilities and Cognitive Tests: What We've Learned from 20 Years of Research. Psychology in the Schools. 2010;47(7):635-50.

30. Blackwell LS, Rodriguez S, Guerra-Carrillo B. Intelligence as a Malleable Construct. In: Goldstein S, Princiotta D, Naglieri JA, editors. Handbook of Intelligence: Evolutionary Theory, Historical Perspective, and Current Concepts. New York, NY: Springer New York; 2015. p. 263-82.

31. Cassidy S, Roche B, Colbert D, Stewart I, Grey IM. A relational frame skills training intervention to increase general intelligence and scholastic aptitude. Learning and Individual Differences. 2016;47:222-35.

32. Løhaugen GCC, Beneventi H, Andersen GL, Sundberg C, Ostgard HF, Bakkan E, et al. Do children with cerebral palsy benefit from computerized working memory training? Study protocol for a randomized controlled trial. Trials. 2014;15(1).

33. Au J, Sheehan E, Tsai N, Duncan G, Buschkuehl M, Jaeggi S. Improving fluid intelligence with training on working memory: a meta-analysis. Psychonomic Bulletin & Review. 2015;22(2):366-77.

34. Jaeggi SM, Buschkuehl M, Jonides J, Perrig WJ. Improving fluid intelligence with training on working memory. Proceedings of the National Academy of Sciences. 2008;105(19):6829-33.

35. Hayes TR, Petrov AA, Sederberg PB. Do we really become smarter when our fluid-intelligence test scores improve? Intelligence. 2015;48:1-14.

For peer review only

For peer review only - http://bmjopen.bmj.com/site/about/guidelines.xhtml

36. Harrison TL, Shipstead Z, Hicks KL, Hambrick DZ, Redick TS, Engle RW. Working Memory Training May Increase Working Memory Capacity but Not Fluid Intelligence. Psychological Science. 2013;24(12):2409-19.

37. Törneke N. Learning RFT : an introduction to relational frame theory and its clinical applications
Learning RFT an introduction to relational frame theory and its clinical applications. Ebooks C, ProQuest, editors: Oakland, CA : Context Press; 2010.

38. Rudd L, Lambert M, Satterwhite M, Zaier A. Mathematical Language in Early Childhood Settings: What Really Counts? Early Childhood Education Journal. 2008;36(1):75-80.

39. Kaufman AS, Raiford SE, Coalson DL. Intelligent testing with the WISC-V. Hoboken, NJ, US: John Wiley & Sons Inc; 2016. xviii, 814-xviii, p.

40. Thirus J, Starbrink M, Jansson B. Relational frame theory, mathematical and logical skills: A multiple exemplar training intervention to enhance intellectual performance. International Journal Of Psychology &Amp. 2016;16(2):141-55.

41. Colbert D, Tyndall I, Roche B, Cassidy SJJoBE. Can SMART Training Really Increase Intelligence? A Replication Study. 2018;27(4):509-31.

42. Wechsler D. WISC-V: Technical and interpretive manual. Bloomington: Pearson; 2014.

43. Wechsler D. Wechsler Intelligence Scale for Children - Fifth Edition Australian and New Zealand Administration and Scoring Manual. Sydney: NCS Pearson; 2016.

44. Joshua N, Wilson C, Bendrups N. WIAT-III A&NZ Examiner's Manual. Sydney: NCS Pearson; 2016.

For peer review only - http://bmjopen.bmj.com/site/about/guidelines.xhtml

45. Gioia GA, Isquith PK, Kenworthy L, Barton RM. Profiles of Everyday Executive Function in Acquired and Developmental Disorders. Child Neuropsychology. 2002;8(2):121-37.

46. Goodman R. Psychometric Properties of the Strengths and Difficulties Questionnaire. Journal of the American Academy of Child & Adolescent Psychiatry. 2001;40(11):1337-45.

47. Rutter M, Bailey A, Lord C. The Social Communication Questionnaire. Los Angeles: Western Psychological Services; 2003.

48. Reynolds CR, Kamphaus RW. Behavior Assessment System for Children. Third ed. Bloomington: Pearson; 2015.

49. Conners CK. Conners' Rating Scales - Revised Technical Manual. Toronto: Multi-Health Systems; 1997.

50. Waters E, Davis E, Mackinnon A, Boyd R, Graham HK, Kai Lo S, et al. Psychometric properties of the quality of life questionnaire for children with CP. Developmental Medicine & Child Neurology. 2007;49(1):49-55.

51. Yin Foo R, Guppy M, Johnston LM. Intelligence assessments for children with cerebral palsy: a systematic review. Developmental medicine and child neurology. 2013;55(10):911-8.

52. Muris P, Meesters C, Eijkelenboom A, Vincken M. The self-report version of the Strengths and Difficulties Questionnaire: Its psychometric properties in 8- to 13-year-old non-clinical children. British Journal of Clinical Psychology. 2004;43(4):437-48.

53. Wei T, Chesnut S, Barnard-Brak L, Richman D. Psychometric Analysis of the Social Communication Questionnaire Using an Item-Response Theory Framework: Implications for the Use of the Lifetime and Current Forms. Journal of Psychopathology and Behavioral Assessment. 2015;37(3):469-80.

For peer review only

For peer review only - http://bmjopen.bmj.com/site/about/guidelines.xhtml

54. Benjamini Y, Hochberg Y. Controlling the False Discovery Rate: A Practical and Powerful Approach to Multiple Testing. Journal of the Royal Statistical Society Series B (Methodological). 1995;57(1):289-300.

55. Faul F, Erdfelder E, Lang A-G, Buchner A. G*Power 3: A flexible statistical power analysis program for the social, behavioral, and biomedical sciences. Behavior Research Methods. 2007;39(2):175-91.

For peer review only

For peer review only - http://bmjopen.bmj.com/site/about/guidelines.xhtml

Figure Legends

Figure 1 - Flow Chart of SMART trial design.

For peer review only

For peer review only - http://bmjopen.bmj.com/site/about/guidelines.xhtml

Flow Chart of Study Design

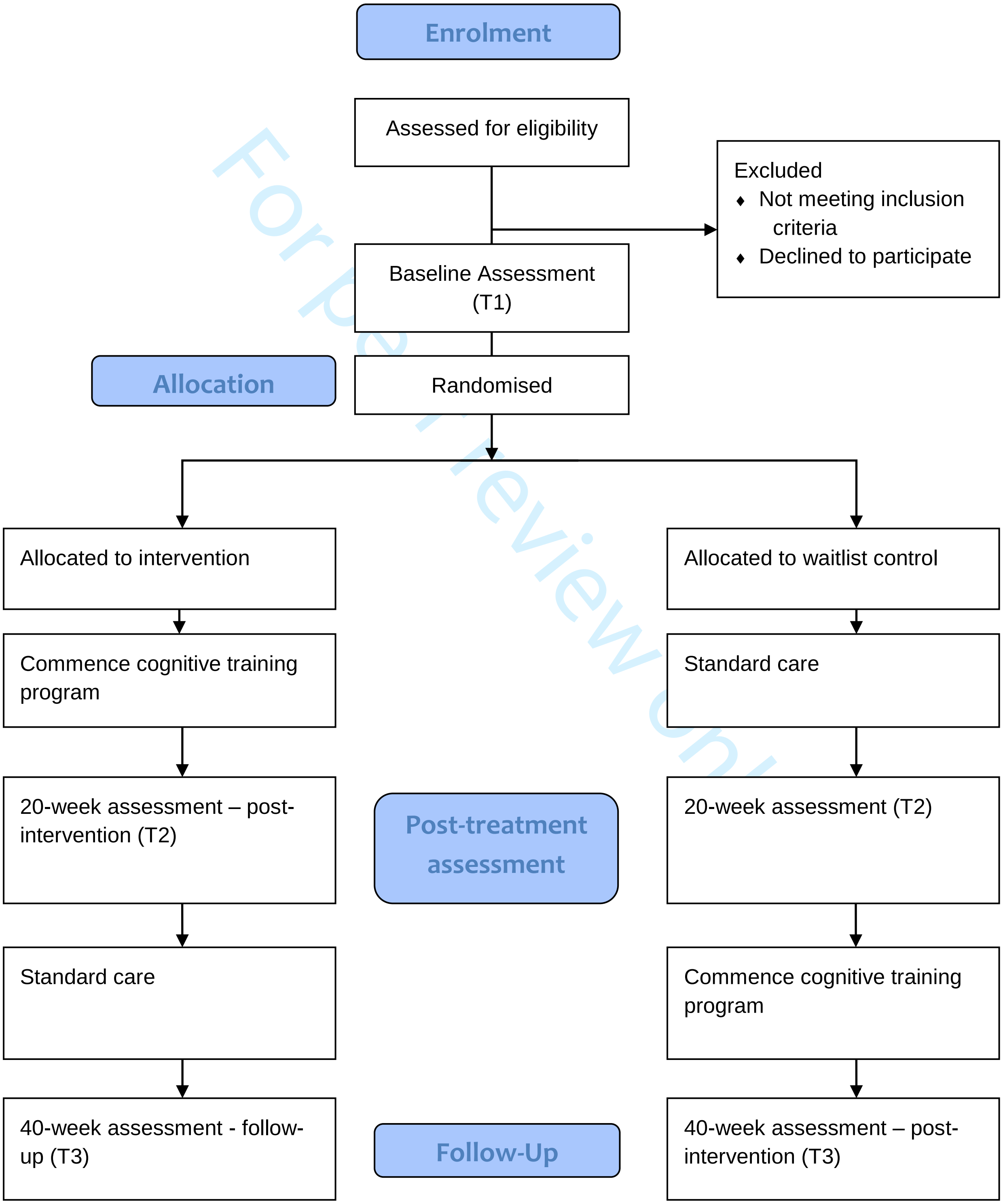

Appendix A

SMART Study Qualitative Interview Questions.

Thank you for taking part in the SMART study. This study has been conducted as part of PhD project through the Queensland Cerebral Palsy and Rehabilitation Research Centre. The study's aim was to independently test the SMART intervention that was originally developed by educational psychologists in Ireland at Maynooth University and determine whether it is effective for children with cerebral palsy.

I would like to ask you about your involvement with SMART to learn more about how other families might engage with the program.

Would it be possible to audio record our conversation so I can prepare an accurate transcript of the discussion?

The recording will be transcribed and stored securely, using your study ID rather than name, to maintain confidentiality.

Parent Questions:

1) What did you like best about the SMART program?

Follow-up: Why did you like X the best?

2) What did you like least about the SMART program?

Follow-up: "Why did you not like X?"

3) What do you think could have been different in the SMART program?

4) Did you experience any challenges in supporting your child in doing SMART?

5) Did you notice any changes in your child after participating in SMART? Follow-up: Have you noticed any changes with regard to schoolwork?

If so, what were the changes you noticed in your child after participating in SMART?

6) Did your child enjoy participating in SMART?

7)Have you noticed any changes in your relationship with your child since participating in SMART?

8) Would you participate in this program again?

9) Would you recommend SMART to other families?

10) Are you planning on continuing to use SMART at home? If so, why? If not, why not?

10) Do you have any further comments or suggestions about using SMART?

Questions for children:

1) What did you like best about the SMART program?

For peer review only

Follow-up: Why did you like X the best?

2) What did you like least about the SMART program?

Follow-up: Why did you not like X?

3) What do you think could have been different in the SMART program?

4) Was there anything that made it easy to use SMART at home? Manual? Parent involvement?

4) Can you tell me about anything that made it hard for you to use SMART at home? Other activities? Time?

5) Did you notice any changes in yourself after participating in SMART? If yes, ask what changes have you noticed after participating in SMART?

6) Did you enjoy participating in SMART?

7) Would you participate in this program again?

9) Would you recommend SMART to other children?

10) Do you think you'd keep using SMART? If so, why? If not, why not?

11) Is there anything else you'd like to tell me about SMART?

For peer review only

Queensland cerebral palsy & rehabilitation research centre

Queensland Government

The University Of Queensland Australia

STANDARD INFORMED CONSENT - CHILD TO PARTICIPATE IN A RESEARCH PROJECT

Project Number

**HREC/14/QRCH/377**

Title of Project

A Randomised Controlled Trial of an Online Cognitive Rehabilitation Program for Children with Cerebral Palsy

Investigator(s)

Dr Koa Whittingham, Dr Jeanie Sheffield, Professor Roslyn Boyd, Ms Jane Wotherspoon

I (Parent/Guardian name) ______________________________

voluntarily consent for my child to take part in the above titled Research Project, explained to me by

Mr/Ms/Dr/Professor ______________________________

Child's Name ______________________________

Address ______________________________

Contact Phone Numbers ______________________________

I have received a Parent/Guardian Information Statement to keep and I believe I understand the purpose, extent and possible effects of my child's involvement

I have been asked if I would like to have a family member or friend with me while the project was explained

I have had an opportunity to ask questions and I am satisfied with the answers I have received

I understand that the researcher has agreed not to reveal results of any information involving my child, subject to legal requirements

If information about this project is published or presented in any public form, I understand that the researcher will not reveal my child's identity

I understand that if I refuse to consent to my child's participation, or if I withdraw my child from the project at any time without explanation, this will not affect my child's access to the best available treatment options and care from Children's Health Queensland.

If you do not wish to participate, **it is okay to say no**

I understand I will receive a copy of this consent form

- **I consent to participate in this research project.**

YES ☐ **NO** ☐

SIGNATURE ______________________________ Date ______________

I have explained the study to the parent/guardian who has signed above, and believe that they understand the purpose, extent and possible effects of their child's involvement in this study.

RESEARCHER'S SIGNATURE ______________________________ Date ______________

Note: All parties signing the Consent Form must date their own signature.

For peer review only - http://bmjopen.bmj.com/site/about/guidelines.xhtml

STANDARD PROTOCOL ITEMS: RECOMMENDATIONS FOR INTERVENTIONAL TRIALS

SPIRIT 2013 Checklist: Recommended items to address in a clinical trial protocol and related documents*

Section/item	Item No	Description
**Administrative information**		
Title	1	Descriptive title identifying the study design, population, interventions, and, if applicable, trial acronym<br>**Page 1.**
Trial registration	2a	Trial identifier and registry name. If not yet registered, name of intended registry<br>**Page 3. This trial is registered with the Australian New Zealand Clinical Trials Registry, Trial ID: ACTRN12617001550392.**
	2b	All items from the World Health Organization Trial Registration Data Set<br>**NA**
Protocol version	3	Date and version identifier<br>**Page 20**
Funding	4	Sources and types of financial, material, and other support<br>**Page 20**
Roles and responsibilities	5a	Names, affiliations, and roles of protocol contributors<br>**Page 1, page 20**
	5b	Name and contact information for the trial sponsor<br>**NA, no sponsor**
	5c	Role of study sponsor and funders, if any, in study design; collection, management, analysis, and interpretation of data; writing of the report; and the decision to submit the report for publication, including whether they will have ultimate authority over any of these activities<br>**NA**
	5d	Composition, roles, and responsibilities of the coordinating centre, steering committee, endpoint adjudication committee, data management team, and other individuals or groups overseeing the trial, if applicable (see Item 21a for data monitoring committee)<br>**NA**
**Introduction**		

For peer review only

Background and rationale	6a	Description of research question and justification for undertaking the trial, including summary of relevant studies (published and unpublished) examining benefits and harms for each intervention
**Page 2, page 4-**		**Page 2, Page 4, page 9**
	6b	Explanation for choice of comparators<br>**Page 6, page 9**
Objectives	7	Specific objectives or hypotheses<br>**Page 9-10**
Trial design	8	Description of trial design including type of trial (eg, parallel group, crossover, factorial, single group), allocation ratio, and framework (eg, superiority, equivalence, noninferiority, exploratory)<br>**Page 10**
**Methods: Participants, interventions, and outcomes**		
Study setting	9	Description of study settings (eg, community clinic, academic hospital) and list of countries where data will be collected. Reference to where list of study sites can be obtained<br>**Page 11**
Eligibility criteria	10	Inclusion and exclusion criteria for participants. If applicable, eligibility criteria for study centres and individuals who will perform the interventions (eg, surgeons, psychotherapists)<br>**Page 11**
Interventions	11a	Interventions for each group with sufficient detail to allow replication, including how and when they will be administered<br>**Page 18**
	11b	Criteria for discontinuing or modifying allocated interventions for a given trial participant (eg, drug dose change in response to harms, participant request, or improving/worsening disease)<br>**NA**
	11c	Strategies to improve adherence to intervention protocols, and any procedures for monitoring adherence (eg, drug tablet return, laboratory tests)<br>**Page 11**
	11d	Relevant concomitant care and interventions that are permitted or prohibited during the trial<br>**NA**

For peer review only - http://bmjopen.bmj.com/site/about/guidelines.xhtml

Outcomes	12	Primary, secondary, and other outcomes, including the specific measurement variable (eg, systolic blood pressure), analysis metric (eg, change from baseline, final value, time to event), method of aggregation (eg, median, proportion), and time point for each outcome. Explanation of the clinical relevance of chosen efficacy and harm outcomes is strongly recommended<br>**Page 13 to page 17**
Participant timeline	13	Time schedule of enrolment, interventions (including any run-ins and washouts), assessments, and visits for participants. A schematic diagram is highly recommended (see Figure)<br>**Flow Chart Figure 1 (CONSORT Flow chart)**
Sample size	14	Estimated number of participants needed to achieve study objectives and how it was determined, including clinical and statistical assumptions supporting any sample size calculations<br>**Page 18-19**
Recruitment	15	Strategies for achieving adequate participant enrolment to reach target sample size<br>**Page 11.**

Methods: Assignment of interventions (for controlled trials)

Allocation:

Sequence generation	16a	Method of generating the allocation sequence (eg, computer-generated random numbers), and list of any factors for stratification. To reduce predictability of a random sequence, details of any planned restriction (eg, blocking) should be provided in a separate document that is unavailable to those who enrol participants or assign interventions<br>**Page 11**
Allocation concealment mechanism	16b	Mechanism of implementing the allocation sequence (eg, central telephone; sequentially numbered, opaque, sealed envelopes), describing any steps to conceal the sequence until interventions are assigned<br>**Page 11**
Implementation	16c	Who will generate the allocation sequence, who will enrol participants, and who will assign participants to interventions<br>**Page 11-12**
Blinding (masking)	17a	Who will be blinded after assignment to interventions (eg, trial participants, care providers, outcome assessors, data analysts), and how<br>**Page 12 (no blinding)**

For peer review only - http://bmjopen.bmj.com/site/about/guidelines.xhtml

	17b	If blinded, circumstances under which unblinding is permissible, and procedure for revealing a participant's allocated intervention during the trial<br>**NA**

Methods: Data collection, management, and analysis

Data collection methods	18a	Plans for assessment and collection of outcome, baseline, and other trial data, including any related processes to promote data quality (eg, duplicate measurements, training of assessors) and a description of study instruments (eg, questionnaires, laboratory tests) along with their reliability and validity, if known. Reference to where data collection forms can be found, if not in the protocol<br>**Page 12, Page 13-17**
	18b	Plans to promote participant retention and complete follow-up, including list of any outcome data to be collected for participants who discontinue or deviate from intervention protocols<br>**Page 18**
Data management	19	Plans for data entry, coding, security, and storage, including any related processes to promote data quality (eg, double data entry; range checks for data values). Reference to where details of data management procedures can be found, if not in the protocol<br>**Page 12**
Statistical methods	20a	Statistical methods for analysing primary and secondary outcomes. Reference to where other details of the statistical analysis plan can be found, if not in the protocol<br>**Page 18**
	20b	Methods for any additional analyses (eg, subgroup and adjusted analyses)<br>**Page 18**
	20c	Definition of analysis population relating to protocol non-adherence (eg, as randomised analysis), and any statistical methods to handle missing data (eg, multiple imputation)<br>**Page 18**

Methods: Monitoring

Data monitoring	21a	Composition of data monitoring committee (DMC); summary of its role and reporting structure; statement of whether it is independent from the sponsor and competing interests; and reference to where further details about its charter can be found, if not in the protocol. Alternatively, an explanation of why a DMC is not needed<br>**Page 12**

For peer review only - http://bmjopen.bmj.com/site/about/guidelines.xhtml

	21b	Description of any interim analyses and stopping guidelines, including who will have access to these interim results and make the final decision to terminate the trial<br>**NA**
Harms	22	Plans for collecting, assessing, reporting, and managing solicited and spontaneously reported adverse events and other unintended effects of trial interventions or trial conduct<br>**Page 12**
Auditing	23	Frequency and procedures for auditing trial conduct, if any, and whether the process will be independent from investigators and the sponsor<br>**NA**
**Ethics and dissemination**		
Research ethics approval	24	Plans for seeking research ethics committee/institutional review board (REC/IRB) approval<br>**Page 19**
Protocol amendments	25	Plans for communicating important protocol modifications (eg, changes to eligibility criteria, outcomes, analyses) to relevant parties (eg, investigators, REC/IRBs, trial participants, trial registries, journals, regulators)<br>**Page 19**
Consent or assent	26a	Who will obtain informed consent or assent from potential trial participants or authorised surrogates, and how (see Item 32)<br>**Page 19**
	26b	Additional consent provisions for collection and use of participant data and biological specimens in ancillary studies, if applicable<br>**NA**
Confidentiality	27	How personal information about potential and enrolled participants will be collected, shared, and maintained in order to protect confidentiality before, during, and after the trial<br>**Page 12**
Declaration of interests	28	Financial and other competing interests for principal investigators for the overall trial and each study site<br>**Page 21**
Access to data	29	Statement of who will have access to the final trial dataset, and disclosure of contractual agreements that limit such access for investigators<br>**Page 20**

For peer review only - http://bmjopen.bmj.com/site/about/guidelines.xhtml

Ancillary and post-trial care	30	Provisions, if any, for ancillary and post-trial care, and for compensation to those who suffer harm from trial participation<br>**NA**
Dissemination policy	31a	Plans for investigators and sponsor to communicate trial results to participants, healthcare professionals, the public, and other relevant groups (eg, via publication, reporting in results databases, or other data sharing arrangements), including any publication restrictions<br>**Page 19**
	31b	Authorship eligibility guidelines and any intended use of professional writers<br>**Page 19**
	31c	Plans, if any, for granting public access to the full protocol, participant-level dataset, and statistical code<br>**NA**
**Appendices**		
Informed consent materials	32	Model consent form and other related documentation given to participants and authorised surrogates<br>**Appendix B**
Biological specimens	33	Plans for collection, laboratory evaluation, and storage of biological specimens for genetic or molecular analysis in the current trial and for future use in ancillary studies, if applicable<br>**NA**

*It is strongly recommended that this checklist be read in conjunction with the SPIRIT 2013 Explanation & Elaboration for important clarification on the items. Amendments to the protocol should be tracked and dated. The SPIRIT checklist is copyrighted by the SPIRIT Group under the Creative Commons "Attribution-NonCommercial-NoDerivs 3.0 Unported" license.

For peer review only - http://bmjopen.bmj.com/site/about/guidelines.xhtml
